# Patient and hospital factors associated with 30-day readmissions after coronary artery bypass graft (CABG) surgery: a systematic review and meta-analysis

**DOI:** 10.1186/s13019-021-01556-1

**Published:** 2021-06-10

**Authors:** Md Shajedur Rahman Shawon, Michael Odutola, Michael O. Falster, Louisa R. Jorm

**Affiliations:** Centre for Big Data Research in Health, University of New South Wales (UNSW) Sydney, Kensington, Australia

**Keywords:** Coronary artery bypass graft, Cabg, Readmission, Hospitalisation, 30-day readmission, Patient factors, Hospital factors

## Abstract

**Background:**

Readmission after coronary artery bypass graft (CABG) surgery is associated with adverse outcomes and significant healthcare costs, and 30-day readmission rate is considered as a key indicator of the quality of care. This study aims to: quantify rates of readmission within 30 days of CABG surgery; explore the causes of readmissions; and investigate how patient- and hospital-level factors influence readmission.

**Methods:**

We conducted systematic searches (until June 2020) of PubMed and Embase databases to retrieve observational studies that investigated readmission after CABG. Random effect meta-analysis was used to estimate rates and predictors of 30-day post-CABG readmission.

**Results:**

In total, 53 studies meeting inclusion criteria were identified, including 8,937,457 CABG patients. The pooled 30-day readmission rate was 12.9% (95% CI: 11.3–14.4%). The most frequently reported underlying causes of 30-day readmissions were infection and sepsis (range: 6.9–28.6%), cardiac arrythmia (4.5–26.7%), congestive heart failure (5.8–15.7%), respiratory complications (1–20%) and pleural effusion (0.4–22.5%). Individual factors including age (OR per 10-year increase 1.12 [95% CI: 1.04–1.20]), female sex (OR 1.29 [1.25–1.34]), non-White race (OR 1.15 [1.10–1.21]), not having private insurance (OR 1.39 [1.27–1.51]) and various comorbidities were strongly associated with 30-day readmission rates, whereas associations with hospital factors including hospital CABG volume, surgeon CABG volume, hospital size, hospital quality and teaching status were inconsistent.

**Conclusions:**

Nearly 1 in 8 CABG patients are readmitted within 30 days and the majority of these are readmitted for noncardiac causes. Readmission rates are strongly influenced by patients’ demographic and clinical characteristics, but not by broadly defined hospital characteristics.

**Supplementary Information:**

The online version contains supplementary material available at 10.1186/s13019-021-01556-1.

## Background

The annual volume and population-based rate of coronary artery bypass graft (CABG) surgery have declined significantly over the past two decades in the United States of America (USA) and in other developed countries [1–4]. These declines reflect lower incidence of coronary artery disease [5] and increased use of percutaneous coronary artery interventions (PCI) instead of CABG [1, 2]. Patients undergoing CABG in recent years also tend to have more extensive disease and more comorbidities than previously [1]. However, CABG is still the most common cardiac surgical procedure in the USA, with 156,931 procedures performed in 2016 [6].

Unplanned readmissions following coronary artery bypass graft (CABG) surgery are associated not only with poorer outcomes (including increased mortality) for patients but also with significant health care costs for payers and patients [7–9]. While reduction of unplanned readmissions in patients undergoing CABG is a clinical priority, the 30-day risk-standardised unplanned readmission rate following CABG is considered in the Hospital Readmissions Reduction Program (HRRP) in the USA, which penalizes hospitals financially for above-expected rates [10]. Given these significant clinical and policy implications, it is important to identify and address factors driving unplanned readmissions following CABG.

For achieving the goal of preventing post-CABG readmissions, effective and well-coordinated patient care interventions (such as telemonitoring, cardiac rehabilitation, patient education, and follow-up appointments) [11] are needed. Identifying the underlying causes of readmission can highlight which care processes should be the focus of attention and effort, whereas examining the patient-level factors associated with readmission can help to identify patient groups to target for improved inpatient care and post-procedure follow-up. Because substantial between-hospital variation in post-CABG readmission rate has been reported previously [12–14] and policies on patient safety and quality of care are usually implemented at the hospital level, certain hospital characteristics may also need to be targeted. To date, no study has systematically collated the evidence regarding the causes of post-CABG readmissions and patient-level as well as hospital-level characteristics associated with such readmissions.

In this systematic review and meta-analysis of 30-day unplanned readmissions after CABG, our key aims were to: (1) quantify rates of unplanned readmission within 30 days of CABG surgery; (2) examine how these readmission rates vary according to different study-level characteristics; (3) explore the underlying causes of 30-day unplanned readmissions following CABG; and (4) investigate associations of various patient- and hospital-level factors with 30-day readmission following CABG.

## Methods

### Data sources and search strategies

This review was conducted in accordance with PRISMA guidelines (see Additional File 1) [15]. Two electronic databases (PubMed and EMBASE) were searched up until June 2020, without any restrictions on language, publication date, source of study population or study size. We searched for published studies with combinations of relevant search terms as outlined in Additional File 2. We also searched cited references in the included papers for further relevant papers.

### Study selection

We included studies identified by the systematic search in the review if they met all of the following criteria: (1) study population: adult patients undergoing CABG, irrespective of indication, severity of disease, and whether carried out as an isolated procedure or in combination with other cardiac surgeries; (2) study design: observational studies; (3) outcome: hospital readmissions within 30 days of CABG surgery, irrespective of cause of readmission; (4) comparison or control group: none; (5) article type: original research articles published in peer-reviewed journals; and (6) language: written in English.

We excluded studies if they (1) did not report CABG-specific readmissions; (2) were restricted to special study populations (e.g., patients undergoing dialysis); (3) were intervention studies or had only matched analysis; and (4) were review articles, or meeting or conference papers.

Using these selection criteria, two independent reviewers (MS and MO) screened titles and abstracts of all studies initially identified through the systematic search. Any disagreement was resolved through consensus. Full texts for further evaluation were retrieved for studies that satisfied all selection criteria. Details of quality assessment is given in supplementary methods.

### Data extraction

We extracted the following information from each included study: authors, year of publication, country, study period, data source, sample size, mean age at CABG procedure, proportion of male patients, proportions of patients with diabetes, hypertension, dyslipidemia, renal failure, heart failure, elective procedure, and isolated procedure. We defined data source of a study as either “administrative data” if data were generated through the routine administration of health care programs or as “medical records data” if data were derived from medical records designed to support individual patient care, whether electronic or not. We also extracted estimates of post-CABG 30-day readmission rates, causes of readmission, and adjusted odds ratios (ORs) with 95% confidence intervals (CIs) for the associations of various patient-level and hospital-level factors with 30-day readmission rates. In instances of multiple studies based on the same data, the most up-to-date or the most comprehensive results were extracted.

### Quality assessment of included studies

Quality assessment of the included studies was conducted independently by two reviewers (MS and MO) using the Newcastle-Ottawa Scale (NOS) [16] for cohort studies using observational data (see Additional File 3). This scale awards a maximum of nine points to each study using three dimensions for quality assessment: selection (up to 4 points), comparability (up to 2 points), and assessment of outcome (up to 3 points) [16]. We categorized study quality based on the total score: low (0–3), moderate [4–6], and high [7–9]. The “comparability of cohorts” criterion was deemed to be met if the study used a multicenter or national database, and “adequacy of follow-up of cohorts,” was deemed acceptable if a study accounted for readmissions to both index and non-index hospitals [17, 18].

### Statistical analysis

We calculated summary estimates for readmission rates within 30 days after CABG by pooling the study-specific estimates using random-effects models to allow for between study heterogeneity, using the *“metaprop”* program in Stata v16.0 [19]. I^2^ statistic was used to estimate the variation in the estimates attributable to between-study heterogeneity, while between-study variance was estimated by τ^2^. We also estimated 30-day readmission rates after CABG according to prespecified study-level characteristics (publication year, country, data source, study size, study quality, proportion of elective procedures and inclusion of isolated CABG patients only). Differences between these group-specific readmission rates were assessed by tests for between-subgroup heterogeneity (P _Heterogeneity_ < 0.05 indicated significant difference between groups). We did narrative synthesis for the causes of 30-day readmissions after CABG because pooling those estimates quantitatively did not seem appropriate.

For the associations of patient-level factors (e.g., sociodemographic and comorbidities) with 30-day readmission rates, we used the inverse variance weighted method to combine study-specific ORs (with 95% CIs) using random-effects models in the *“meta”* program of Stata v16.0. Between-study heterogeneity was assessed using the Cochrane χ^2^ statistic and the I^2^ statistic. To assess the associations between hospital-level factors and 30-day readmission rates, we constructed descriptive summary tables because those estimates could not be quantitatively pooled.

## Results

### Study identification and selection

**Fig. 1:**
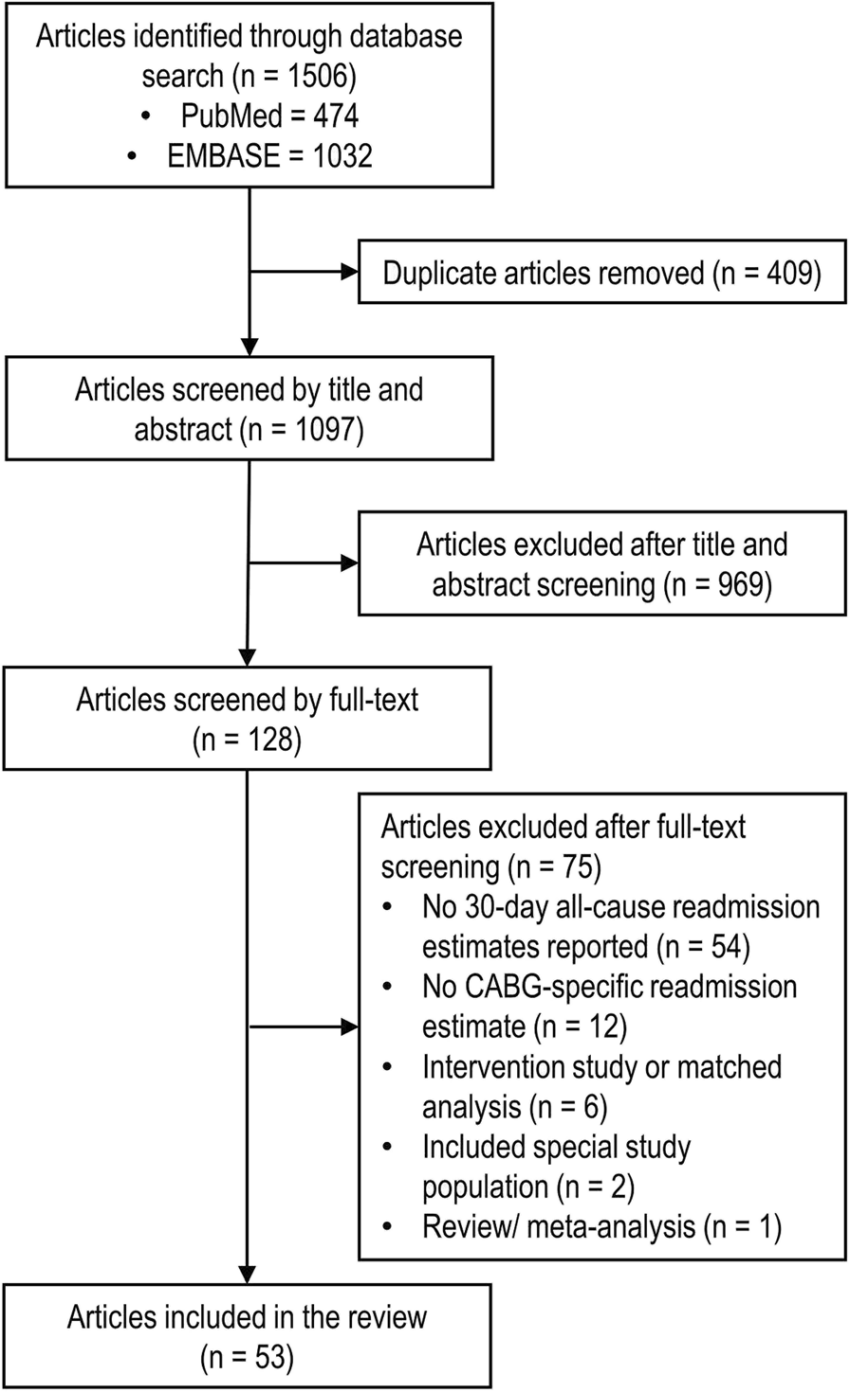
PRISMA flow diagram for study selection in the systematic review

We identified 1506 relevant citations. After screening titles and abstracts, 128 articles were selected for full text retrieval and detailed evaluation. As shown in Fig. 1, after full-text assessment, 75 studies were excluded. The remaining 53 studies were included in the systematic review and relevant meta-analyses.

### Characteristics of the included studies

Characteristics of all 53 studies [7, 8, 11–14, 20–66] included in this systematic review are given in Table 1. Forty studies [7, 8, 11–14, 20–26, 28–37, 39–44, 46–49, 52, 54, 58, 59, 62, 64, 65] (75% of all included studies) were from the USA. Most studies [7, 8, 11–14, 20, 21, 25, 27, 32, 33, 35–37, 39–42, 44, 46–49, 52, 58, 59, 61, 63, 65, 66] (*n* = 31) were based on administrative data aggregated across multiple sites and 22 studies [22–24, 26, 28–31, 34, 38, 43, 45, 50, 51, 53–57, 60, 62, 64] used medical records from either a single center or multiple centers. The included studies varied widely in sample size (range: 110 to 1,116,991). Most participants in all studies were male (range 65 to 99%). The mean age at CABG procedure ranged from 57 years to 76 years. In studies where history of comorbidities was reported, diabetes (range: 12 to 69%) and hypertension (range: 37 to 95%) were relatively common in patients undergoing CABG. While most studies included patients, who had either isolated or combined CABG procedures, 22 studies [8, 13, 26, 29, 31, 33, 35–37, 39, 40, 45–48, 50, 51, 54, 56, 58, 59, 62] included only those who underwent isolated CABG procedures. The included studies varied widely (range: 15 to 100%) in the proportion of elective procedures (Table 1).

**Table 1:** Characteristics of the included studies assessing 30-day readmissions after CABG

Author’s last name (Year), Country	Data source (Study period)	Study sample	% Male	Mean age at procedure	% Diabetes	% Hypertension	% Elective procedure	% Isolated procedure
Alkhouli (2019) [20], USA	National Readmissions Database (2015–2016)	411,159	75%	66 years	51%	84%	50%	
Anderson (2016) [21], USA	California Coronary Artery Bypass Grafting Outcomes Reporting Program (2011–2012)	21,638						
Angraal (2018) [12], USA	Medicare fee-for-service inpatient claims data (1999–2004)	1,863,719	66%	74 years	31%	63%		
Auerbach (2009) [22], USA	Clinical data from multiple centres in North Carolina (2003–2005)	81,289	72%	65 years	37%	72%		
Barnett (2018) [23], USA	Clinical data from multiple centres (2008–2011)	5818	99%	60 years	49%		100%	
Benuzillo (2018) [24], USA	Clinical data from multiple centres (2010–2014)	2589	80%	67 years	42%	83%	43%	
Bianco (2019) [25], USA	Pennsylvania Health Care Cost Containment Council databases (2014–2016)	16,641						
Bianco (2019) [26], USA	Clinical data from a single centre (2011–2017)	7048	75%	65 years	46%	90%	42%	100%
Blackledge (2009) [27], UK	Hospital inpatient data (1995–2004)	2520	79%		21%		82%	
Brooke (2015) [7], USA	Medicare and Medicaid beneficiary data (2001–2011)	1,502,815						
Case (2019) [28], USA	Clinical data from a single centre (2011–2017)	150	75%	63 years	49%		63%	
Chan (2020) [29], USA	Clinical data from a single centre (2011–2017)	4980	75%	66 years	47%	90%	34%	100%
Chen (2015) [30], USA	Patient discharge database (2011)	5813	77%	67 years				
Cho (2019) [31], USA	Clinical data from a single centre (2013–2016)	1552	75%	65 years	29%	80%		100%
Connolly (2018) [32], USA	Florida, California, New York, Maryland, and Kentucky State Inpatient Databases (2007–2014)	312,018	74%	66 years	30%	58%		
Deo (2019) [33], USA	National readmission database (2014)	135,699	75%	66 years	46%			100%
Fanari (2017) [34], USA	Clinical data from multiple centres (2010–2013)	1277	74%	67 years	44%		49%	
Feng (2018) [35], USA	California, Florida, and New York State Inpatient Databases (2007–2011)	177,229	74%	66 years	41%	80%		100%
Fox (2013) [36], USA	California State Inpatient and Emergency Department Databases (2005–2009)	63,911	75%					100%
Girotti (2014) [37], USA	National Medicare beneficiaries database (2006–2008)	232,980	68%	74 years	29%	65%	48%	100%
Gurram (2019) [38], India	Clinical data from a single centre (2015–2016)	773	83%	62 years	73%	71%		
Hannan (2003) [39], USA	New York state inpatient database (1999)	16,325	72%		32%			100%
Hannan (2011) [40], USA	New York state inpatient database (2005–2007)	33,936	74%		36%			100%
Hirji (2020) [41], USA	National readmission database (2010–2015)	844,206	67%					
Hwang (2007) [42], USA	5% sample of Medicare beneficiaries database (2001–2003)	22,091	65%	All patients aged 65+ years	27%	60%	48%	
Iribarne (2014) [43], USA	Clinical data from multiple centres in the United States and Canada (2010)	5059	67%	64 years	23%		76%	33%
Khuory (2019) [8], USA	Nationwide readmission database (2010–2014)	855,836	75%	65 years	47%	80%		100%
Kim (2015) [44], USA	Administrative claims database (2010–2012)	41,031						
Koochmeshki (2013) [45], Iran	Clinical data from a single centre (2004–2011)	952	70%	59 years	23%	40%		100%
Lancey (2014) [46], USA	Society of Thoracic Surgeons (STS) compliant registry (2007–2011)	4861	73%	65 years	40%	84%		100%
Li (2012) [48], USA	California CABG outcomes reporting program (2009)	11,823	75%	57% aged > 65 years	45%	87%	40%	100%
Li (2014) [47], USA	California CABG outcomes reporting program (2010–2011)	22,389	76%	22% aged > 75 years	46%	87%	41%	100%
Li (2015) [49], USA	California CABG outcomes reporting program (2012)	14,051	75%	67 years	52%	88%	40%	83%
McNeely (2017) [13], USA	Medicare and Medicaid Services data (2000–2012)	1,116,991	68%	74 years	30%	60%	32%	100%
Narain (2019) [50], UK	Clinical data from a single centre (2012–2017)	649	83%	67 years	31%	80%	50%	100%
O’Brien (2018) [51], Australia	Australian and New Zealand Society of Cardiac and Thoracic Surgeons registry (2000–2012)	36,902	79%	65 years	35%	80%		100%
Price (2013) [52], USA	New York Cardiac Surgery Reporting System (CSRS) (2006–2011)	1205	77%	65 years	31%			
Reis (2008) [53], Brazil	Clinical data from a single centre (2006–2007)	290			29%	79%		
Rosenblum (2019) [54], USA	Clinical data from a single centre (2002–2017)	21,719		63 years	41%	86%	64%	100%
Saab (2013) [55], Lebanon	Clinical data from a single centre (2010)	110	86%	50% aged > 65 years	43%	76%		
Saito (2019) [56], Japan	Japan Cardiovascular Surgery Database (2015–2016)	29,395	79%		54%	77%		100%
Sargin (2016) [57], Turkey	Clinical data from a single centre (2013)	1103	55%	65 years	26%	69%		
Sedrakyan (2016) [14], USA	Register data from New York and California states (2005–2011)	198,461	72%	29% aged > 75 year	42%	63%		
Shah (2019) [58], USA	National readmission database (2013–2014)	288,059	75%	65 years	32%	73%	46%	100%
Shahian (2014) [59], USA	Society of Thoracic Surgeons National Database (2008–2010)	162,572	69%	69% aged > 70 year	40%	87%		100%
Shehata (2013) [60], Canada	Clinical data from a single centre (2007–2009)	2102	77%	66 years		65%		
Slamowicz (2008) [61], Australia	Victorian Admitted Episodes Dataset (1998–2003)	6627	78%	65 years			100%	
Stewart (2000) [62], USA	Clinical data from a single centre (1997)	485	67%	65 years	37%	62%		100%
Tam (2018) [63], Canada	CorHealth Ontario Cardiac Registry (2008–2016)	63,336	76%	66 years	34%	71%	62%	
Trooboff (2019) [64], USA	Clinical data from multiple centres (2008–2010)	1683						78%
Tsai (2013) [65], USA	National Medicare data (2009–2010)	153,496						
Tseng (2018) [66], Taiwan	Health insurance database (2005)	1575	93%	65 years				
Zywot (2018) [11], USA	Hospital Readmission reduction database from California and New York (2006–2011)	126,519	71%	34% aged 75+ years	41%	77%		

### 30-day readmission rates after CABG

**Fig. 2:**
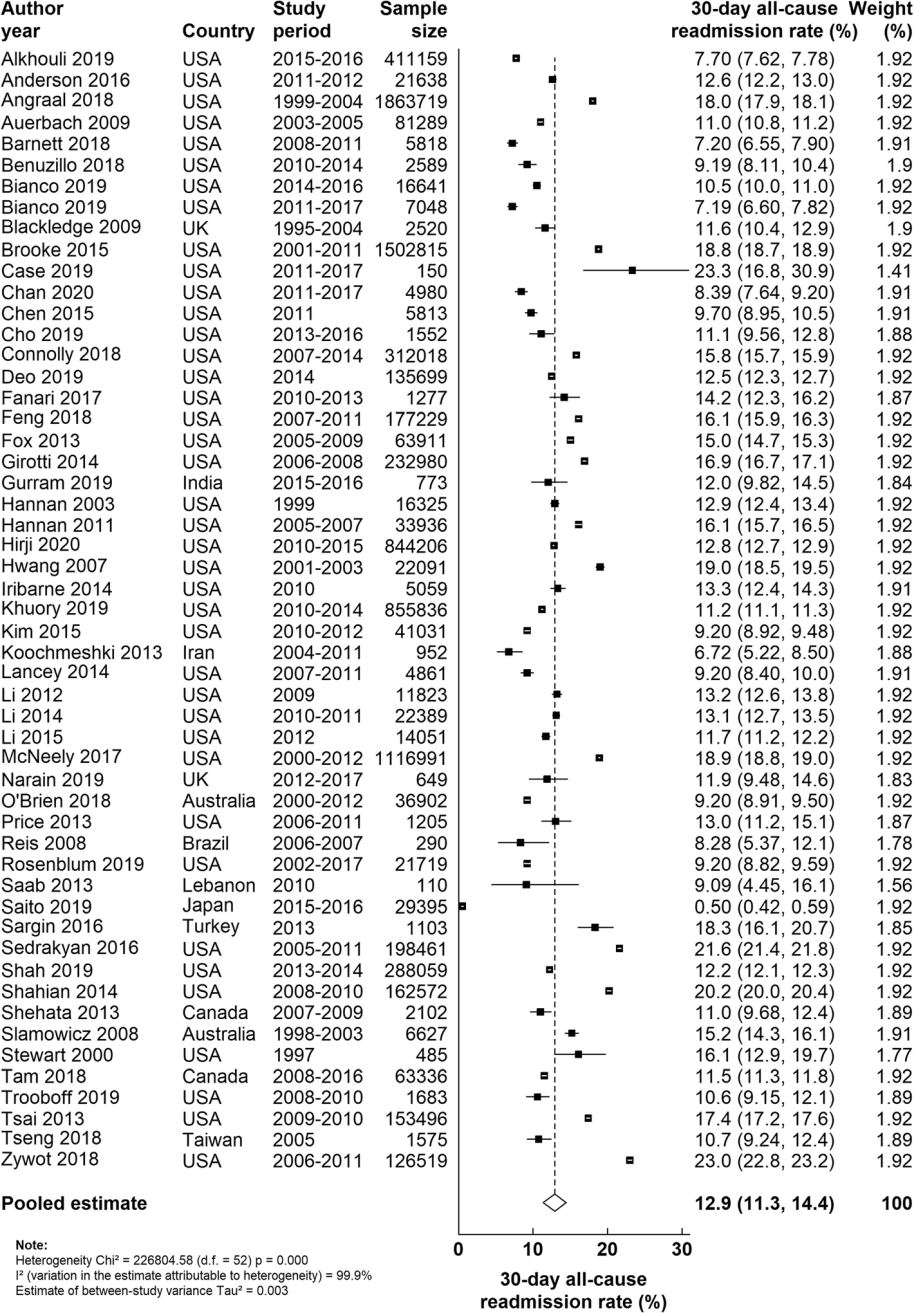
Random-effect meta-analysis for 30-day readmission rate after CABG. Individual study-specific estimates and their 95% CIs are indicated by the black squares and the horizontal lines, respectively

**Fig. 3:**
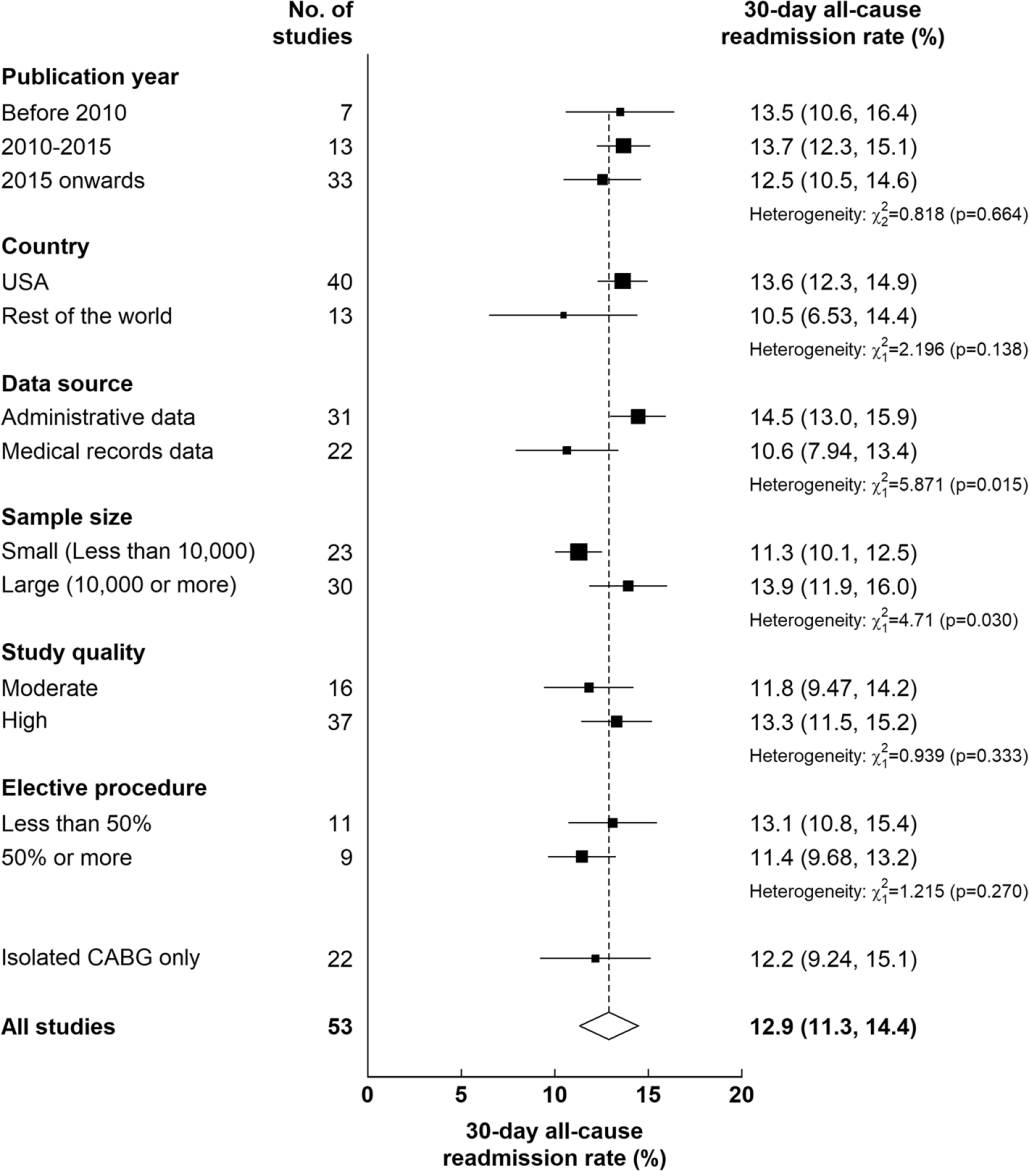
Subgroup analysis of 30-day readmission rate after CABG by various study characteristics. The black squares and the horizontal lines indicate pooled estimates from random effect meta-analysis of studies belong to a specific subgroup and the corresponding 95% CIs, respectively. Heterogeneity chi-squares are based on tests of heterogeneity between subgroups

Among individual studies, 30-day readmission rates following CABG ranged from 0.50% in Saito et al. [56] to 23.3% in Case et al. [28] The pooled 30-day readmission rate after CABG was 12.9% (95% CI: 11.3–14.4%) (Fig. 2). In terms of study characteristics, we found evidence of significant between-subgroup heterogeneity in the pooled 30-day readmission rate by data source (administrative data vs medical record data: 14.5% vs 10.6%; *P* = 0.015) and by study size (large studies [≥10,000 patients] vs small studies [< 10,000 patients]: 13.9% vs 11.3%, *P* = 0.03) (Fig. 3). Although 30-day readmission rates varied when studies were grouped by publication year, country, study quality and proportion of elective procedure, no statistically significant between-subgroup heterogeneity was observed for these study-level characteristics (Fig. 3). When restricting to studies that included only patients undergoing isolated CABG, the 30-day readmission rate did not change appreciably (12.2%).

Information on the readmission destination (index vs. non-index hospitals) after CABG was available from only three US studies (see additional file 4) [7, 41, 48]. Around one-third (range: 27.3 to 34.6%) of all patients readmitted within 30 days after CABG were admitted to non-index hospitals.

### Causes of 30-day readmission after CABG

**Fig. 4:**
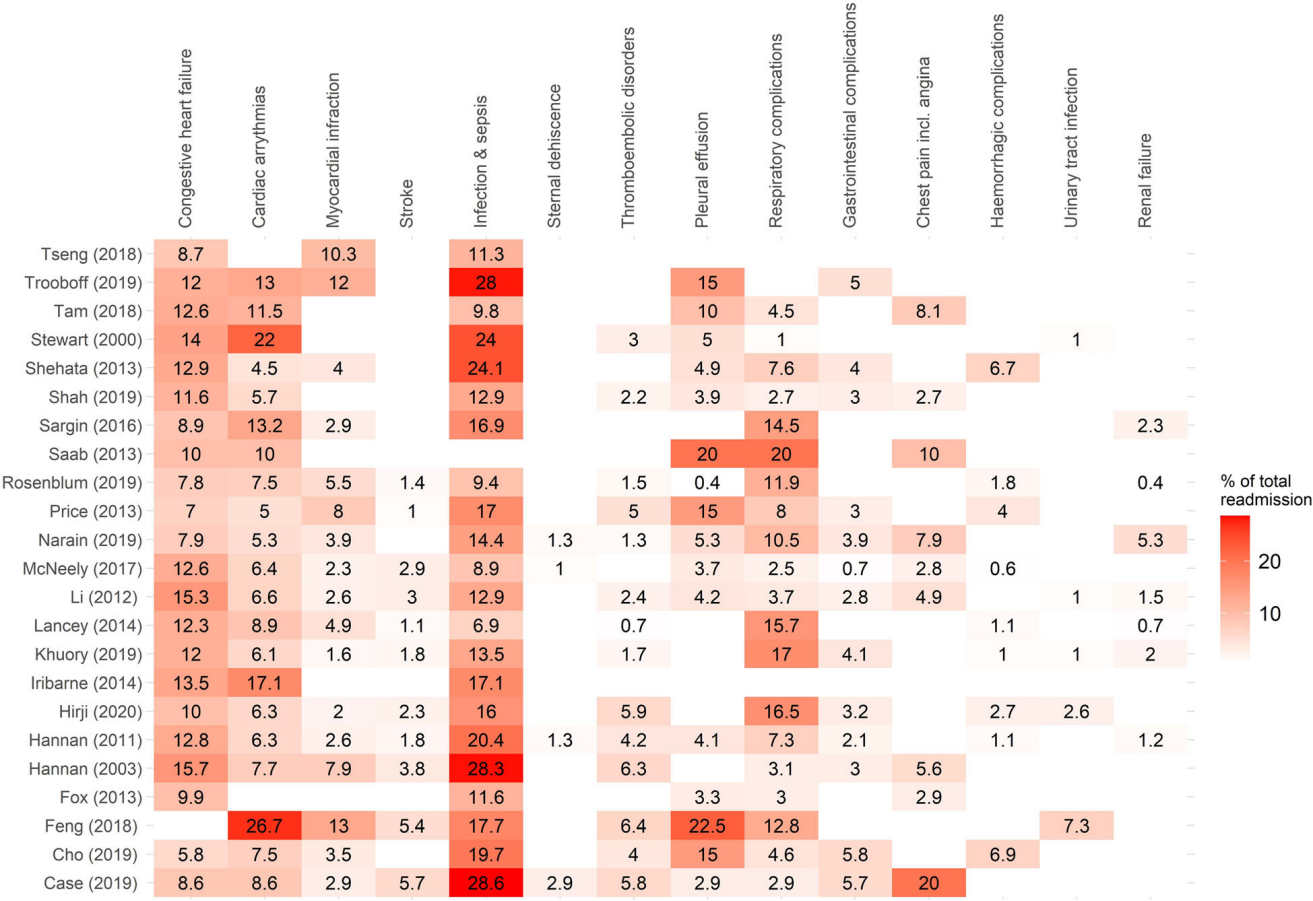
Causes of 30-day readmission after CABG

Twenty-three studies [8, 13, 28, 31, 35, 36, 39–41, 43, 46, 48, 50, 52, 54, 55, 57, 58, 60, 62–64, 66] reported the causes of 30-day readmission after CABG (Fig. 4). Cardiac causes, most frequently congestive heart failure and arrythmias, constituted between 10 and 40% of all readmissions. Between 6.9 and 28.6% of all readmissions within 30 days after CABG were due to infection and sepsis (Fig. 4). Other commonly reported causes of 30-day readmission were pleural effusion (range: 5 to 23.3%), respiratory complications (1 to 20%), thromboembolic disorders (0.7 to 6.3%), and gastrointestinal complications (0.7 to 5.8%) (Fig. 4).

### Patient and hospital factors associated with 30-day readmission

**Fig. 5:**
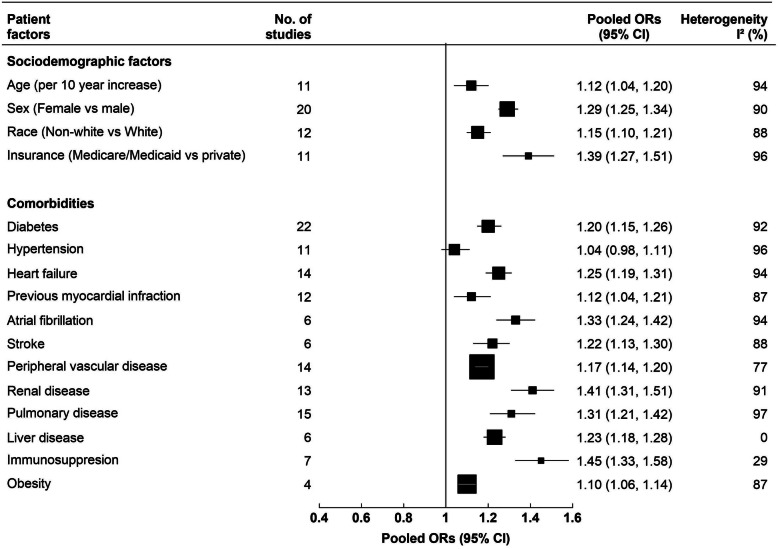
Pooled odds ratios (ORs) with 95% confidence intervals (CIs) from random effect meta-analysis for associations between various patient factors and 30-day readmission after CABG. The black squares and the horizontal lines indicate pooled ORs from random effect meta-analysis of studies investigating the association of 30-day readmission with a specific patient factor and the corresponding 95% CIs, respectively. Individual meta-analysis for each variable presented here can be found in S1-S15 Figures in S2 File

Figure 5 presents associations of various patient-level factors with 30-day readmission rate following CABG (Fig. 5). The estimated pooled OR for per 10-year increase in age from 11 studies [24, 25, 28, 34, 35, 39, 47, 59–61] was 1.12 (1.04–1.20). Female sex (pooled OR from 20 studies [11, 13, 25, 26, 28, 33–36, 39, 40, 47–50, 58, 59, 61–63]: 1.29 [1.25–1.34]) and non-White race (pooled OR from 12 studies [8, 11, 13, 28, 34, 35, 37, 39, 40, 47, 48]: 1.15 [1.10–1.21]) were associated with higher risk of readmissions within 30 days after CABG. Compared to those with private insurance, those with Medicare or Medicaid in the US were more likely to be readmitted (pooled OR from 11 studies [8, 11, 32–36, 40, 48, 58]: 1.39 [1.27–1.51]) (Fig. 5).

For meta-analyses for the associations of 12 comorbid conditions with readmission rate after CABG (Fig. 5), data came from 22 studies for diabetes [8, 13, 24–26, 28, 31, 34–36, 39, 40, 46–48, 50, 54, 58, 59, 62, 63], 11 studies for hypertension [8, 13, 35, 36, 47, 48, 54, 59, 62, 63], 14 studies for heart failure [13, 24, 34–36, 39, 40, 47, 48, 50, 58, 59, 62, 63], 12 studies for previous myocardial infarction (MI) [8, 13, 24–26, 28, 34, 39, 48, 54, 62, 63], 6 studies for atrial fibrillation [8, 13, 26, 36, 47, 48], 6 studies for stroke [13, 40, 47, 50, 54, 59], 14 studies for peripheral vascular disease [8, 13, 35, 36, 39, 40, 47, 48, 50, 54, 59, 62, 63], 13 studies for renal disease [8, 13, 28, 31, 35, 36, 39, 40, 48, 50, 58, 63], 15 studies for pulmonary disease [8, 13, 31, 35, 39, 40, 46–48, 50, 54, 58, 59, 62, 63], 6 studies for liver disease [8, 35, 36, 39, 40, 58], 7 studies for immunosuppression [25, 26, 40, 47, 48, 54, 59], and 4 studies for obesity [8, 34, 35, 63]. All of these comorbidities, except hypertension, were found to be significantly associated with 30-day readmission after CABG (Fig. 5). Meta-analyses for each of these comorbidities are given in Figures S1-S16 in Additional File 5. Pooled ORs for 30-day readmission after CABG were highest for immunosuppression (1.45 [1.33–1.58]), renal disease (1.41 [1.31–1.51]), and atrial fibrillation (1.33 [1.24–1.42]) (Fig. 5). Because of the differences in the comparison groups, we could not conduct meta-analysis for the association between comorbidity indices and 30-day readmission after CABG. However, qualitative review suggested that higher scores in either Charlson Comorbidity index or Elixhauser Comorbidity index were associated with higher likelihood of 30-day readmission after CABG (S4 Table) [8, 30, 31, 44, 58, 60, 61, 63, 66].

For the associations of various hospital-level factors and 30-day readmission after CABG, we did not perform meta-analysis due to either differences in comparison groups or limited numbers of studies examining any specific association. A total of eight studies [8, 13, 20, 22, 23, 30, 35, 48] examined the association with hospital CABG volume. Five studies [8, 13, 20, 22, 35] found that lower hospital CABG volume was significantly associated with higher rates of readmission while the other three studies [23, 30, 48] found no significant association (Table 2). For surgeon CABG volume, Hannan et al. [39] reported that the OR comparing ≤100 cases vs > 100 cases was 1.16 (1.03–1.31). However, Auerbach et al. [22] reported no significant association between surgeon CABG volume and CABG readmission. There was no strong evidence for associations of hospital quality indicators (e.g. risk-adjusted mortality rate) [39, 40, 42], number of hospital beds [48, 58], and teaching status [30, 48, 58] with readmission rate after CABG (Table 2).

**Table 2:** Systematic review of hospital factors associated with 30-day readmission after CABG

Author (Year)	Comparison groups	Findings^**a**^
**Annual CABG volume**		
Alkhouli (2019) [20]	Low (< 100)	1.77 (1.68–1.87)
	Intermediate (100–250)	1.11 (1.07–1.14)
	High volume (> 250)	1.00 (Reference)
Auerbach (2009) [22]	Lowest quartile	1.22 (1.06–1.41)
	Q2	1.16 (1.01–1.33)
	Q3	1.21 (1.05–1.39)
	Highest quartile	1.00 (Reference)
Barnett (2018) [23]	Low volume (< 200 cases)	0.83 (0.64–1.09)
	Standard volume (≥200 cases)	1.00 (Reference)
Chen (2015) [30]	Lowest quartile	1.08 (0.66–1.75)
	Q2	1.14 (0.80–1.63)
	Q3	1.07 (0.78–1.46)
	Highest quartile	1.00 (Reference)
Feng (2018) [35]	Lowest quartile	1.00 (Reference)
	Q2	0.94 (0.91–0.98)
	Q3	0.94 (0.90–0.98)
	Highest quartile	0.95 (0.92–0.99)
Khuory (2019) [8]	Lowest tertile	1.00 (Reference)
	Middle tertile	0.93 (0.89–0.96)
	Highest tertile	0.95 (0.91–0.99)
Li (2012) [48]	100–299	1.10 (0.93–1.29)
	≥300	0.99 (0.79–1.22)
	< 100	1.00 (Reference)
McNeely (2017) [13]	< 50	1.17 (1.12–1.21)
	500–100	1.06 (1.02–1.09)
	101–200	1.02 (0.99–1.06)
	> 200	1.00 (Reference)
Tsai (2013) [65]		Adjusted 30-day readmission rates:
	Lowest quartile	19.2%
	Q2	17.4%
	Q3	17.2%
	Highest quartile	17.2%
**No. of hospital bed**		
Li (2012) [48]	≥300	1.04 (0.79–1.36)
	150–299	1.02 (0.78–1.35)
	< 150	1.00 (Reference)
Shah (2019) [58]	Small	0.95 (0.87–1.04)
	Medium	1.00 (0.95–1.06)
	Large	1.00 (Reference)
**Surgeon CABG volume**		
Auerbach (2009) [22]	Lowest quartile	1.02 (0.94–1.11)
	Q2	1.05 (0.96–1.15)
	Q3	1.03 (0.95–1.12)
	Highest quartile	1.00 (Reference)
Hannan (2003) [39]	< 100 cases vs > 100 cases	1.16 (1.03–1.31)
**Hospital teaching status**		
Chen (2015) [30]	Yes, vs no	0.92 (0.63–1.34)
Li (2012) [48]	Yes, vs no	1.12 (0.90–1.35)
Shah (2019) [58]	No vs yes	1.02 (0.97–1.07)
**Hospital quality**		
Hannan (2003) [39]	Hospital risk-adjusted mortality rate, highest tertile vs lowest tertile	1.14 (1.03–1.25)
Hannan (2011) [40]	Hospital risk-adjusted mortality rate, highest tertile vs lowest tertile	1.10 (0.91–1.33)
Hwang (2007) [42]	Hospital degree of cardiac specialization	
	Most	0.96 (0.77–1.19)
	Moderate	1.0 (0.88–1.13)
	Least	1.00 (reference)
Tsai (2013) [65]	Hospital Quality Alliance surgical score	30-day readmission rate:
	Lowest quartile	17.8%
	Q2	17.4%
	Q3	17.2%
	Highest quartile	17.5%

^a^ Findings are presented as adjusted ORs (95% CI), unless otherwise indicated

## Discussion

We conducted a systematic review and meta-analysis to synthesize available evidence on 30-day readmissions after CABG and to understand the relevant clinical and policy implications. Overall, nearly 1 in 8 patients undergoing CABG are readmitted for any cause within 30 days of the procedure. The pooled readmission rates were broadly similar when studies were grouped by various study characteristics. A large proportion of readmissions are due to noncardiac causes such as postsurgical infections and respiratory complications. Taken together, findings from our study suggest that readmission rates are strongly influenced primarily by patients’ demographic characteristics and the presence of comorbidities, whereas we did not identify any broadly defined hospital characteristics that are consistently associated with post-CABG readmissions.

Most of the included studies in this systematic review are from the USA [7, 8, 11–14, 20–26, 28–37, 39–44, 46–49, 52, 54, 58, 59, 62, 64, 65] and we found that these studies had higher rates of 30-day readmission (13.6% vs. 10.5%) than studies conducted outside the USA. Such differences might reflect differences between countries in healthcare systems (e.g., USA has an insurance-based healthcare model whereas many European countries have publicly funded healthcare systems), practice patterns and guidelines for managing acute coronary syndromes, and healthcare resources. We found that the pooled readmission rate for studies based on administrative data was much higher than the pooled readmission rate for studies based on medical records data (14.5% vs. 10.6%). This difference could be explained by the fact that studies using medical records may only be able to track readmissions to the hospital where the initial procedure is performed (i.e., readmissions to the index hospital) whereas administrative data can capture readmissions occurring both to index and other (non-index) hospitals. The three studies in this review that considered re-admission destination (all using administrative data from the USA) reported that nearly one-third of all readmissions within 30 days of CABG occurred in non-index hospitals. Notably, 27 out of 31 studies which are based on administrative data are from USA, so this may at least partly explain the higher 30-day readmission rates observed in USA-based studies. Another source of variation among the USA-based studies could be which database was used to define the CABG patient cohort. Those using the Nationwide Readmission Database captured patients with Medicare, Medicaid, private insurance, and other payers, whereas some studies only included patients with Medicare insurance [67]. Sociodemographic profiles vary between these two databases [68], which might contribute to the observed differences in readmission rates.

We did not observe any appreciable differences in readmission rates by publication year, despite significant changes over time in the risk profile and clinical presentation in patients undergoing CABG, and reductions in post-surgery length of stay [1, 2]. This might be due to the fact that year of publication does not always correlate with year of clinical practice, given some studies covered a large period of time. Two of the included studies [12, 13] using national data for Medicare beneficiaries reported decreasing trends of readmission within 30 days following CABG over the period 1999–2014 while another study [14] reported that the readmission rates did not vary significantly in New York and California states over the period 2005–2011.

Our review suggests that collectively the majority of readmissions after CABG are due to noncardiac causes, including but not limited to infections, pleural effusion, respiratory complications, gastrointestinal complications and bleeding. Since predictors for noncardiac readmissions are more frequently related to system-related factors such as post-discharge care coordination [46], one might argue that certain noncardiac causes of readmissions, for example postoperative infection, should be an important focus for reducing avoidable readmissions. Clinically, it is important to generate evidence regarding whether interventions such as improved care processes, use of discharge checklists, post-discharge care coordination, patient education videos, and early follow-up clinics for high-risk patients can be implemented to reduce these noncardiac causes of readmissions [11]. Patients who undergo CABG often suffer from multimorbidity and managing those comorbidities can potentially prevent a future readmission. Nevertheless, it should be recognized that not all readmissions are preventable. Another reason for focusing on the causes of readmissions is the fact that the potential clinical implications for all readmissions are not the same. For example, Toorboff et al. [64] reported that although infection was the leading primary diagnosis of post-CABG readmissions, nearly three in four patients with sternal infections required a procedure at readmission and only one in four patients with leg infections required a procedure at readmission. On the other hand, the majority of those readmitted for pericardial or pleural effusions required drainage.

Our study confirmed that patients’ sociodemographic (e.g., female sex, older age, non-white race, insurance type) and clinical characteristics (e.g. diabetes, heart failure, previous MI, atrial fibrillation, stroke, peripheral vascular disease, renal disease, pulmonary disease, liver disease, immunosuppression, obesity), rather than hospital characteristics, are the major drivers of readmissions following CABG. Because many of these patient factors are non-modifiable in nature, attention to management of comorbidities at the index hospitalisation as well as close follow-up of high-risk patients (with multiple comorbidities) after discharge may reduce the potentially avoidable readmissions. A previous systematic review reported that similar patient factors were associated with unplanned readmission following PCI [69]. Shared patient-level predictors of unplanned readmissions following CABG and PCI present opportunities for interdisciplinary heart teams to collaborate and improve patient care. We found that there is an inconsistent body of evidence linking various hospital characteristics and post-CABG readmissions. Notably, we found that the association between hospital annual CABG volume and 30-day readmission rate is weak and inconsistent across studies. It has been hypothesized that hospitals with higher CABG volume are likely to have lower readmission rates because they have greater access to experienced surgeons and highly trained staff members, robust preoperative patient care, and optimized postoperative management [8, 20]. Because a significant proportion of readmissions were due to infection, sepsis, and other noncardiac causes, it is likely that patient complexity and other hospital-level characteristics reflecting the care/discharge processes are the major drivers of any hospital variations in 30-day readmission rate. In this review, however, we found that post-CABG readmissions were not consistently related to broadly defined hospital quality indicators or CABG-specific quality of care indicators [22, 39, 40, 42, 65]. More research is needed to better understand the exact drivers of hospital variation in unplanned post-CABG readmissions.

Under the HRRP program in the USA, the Centers for Medicare & Medicaid Services calculates payment reduction for each hospital based on 30-day risk-standardized unplanned readmission rate for six conditions or procedures including CABG [10]. The risk adjustment is done for age, sex, and comorbidity, but according to a recent study large teaching hospitals and safety-net hospitals with bigger shares of vulnerable patients (with low socioeconomic status and more comorbidities) were facing larger penalties in the HRRP program than other hospitals [70]. Accounting for social risk factors to the risk adjustment for readmission rates could reduce the negative unintended consequences for safety-net hospitals [71]. Another competing issue of risk adjustment for readmission rate might be upcoding of the variables included in the risk-adjustment models to game the system [72].

While we conducted the most comprehensive and detailed review to date of post-CABG readmission rates, causes of readmissions, and factors associated with such readmissions, several limitations pertaining to this review and the included studies should be noted. Firstly, we observed a high level of heterogeneity between studies in the meta-analysis of 30-day readmission rate, which warrants cautious interpretations of the pooled estimates. Secondly, while we examined the associations of patient-level and hospital-level factors with 30-day readmission rates, we did not examine the roles of procedural factors (e.g., use of arterial vs venous grafts, harvesting techniques, off-pump vs. on-pump techniques, no. of vessels involved and bypass time) or postoperative factors (e.g., postoperative complications, length of hospital stay and discharge destination) on readmission rates. According to recent studies [46, 50], more than 60% of 30-day readmissions occurred within the first 10 days of discharge. These studies also suggested that earlier readmissions were more likely to be procedure-related than patient-related [46, 50]. Thirdly, regarding the causes of readmissions, individual studies reported the primary reason for unplanned readmissions after CABG, but patients might be readmitted with multiple diagnoses. Administrative data like the National Readmission Database are derived from hospital claims data without access to individual medical records [67]. Therefore, studies based on administrative databases did not have sufficient granularity to answer questions related to clinical presentation or indication for CABG procedure, risk scores, and variation in postoperative outpatient practice patterns, which may further explain readmission rates. Fourthly, administrative databases are also subjected to variations in the diligence and accuracy of data collection across multiple sites. Some studies [39, 40] did not exclude planned or elective readmissions for which the observed rates might be overestimated to some extent. However, Kuhoy et al. [8] reported that only less than 1% of all CABG readmissions in the Nationwide Readmission Database were planned. It is also important to understand that not all readmissions are bad, some are necessary for optimal clinical care [73].

## Conclusions

In conclusion, a significant proportion of patients undergoing CABG require readmissions within 30 days and the majority of these are readmitted for noncardiac causes. 30-day readmission rates are strongly influenced by patients’ demographic and clinical characteristics, but not by broadly defined hospital characteristics. The findings of this study are valuable for benchmarking quality improvement in clinical care as well as informing hospital readmission reduction policies for CABG.

## Supplementary Information

**Additional file 1.** Completed PRISMA checklist.

**Additional file 2.** Search terms used for the systematic review.

**Additional file 3.** Quality assessment of included studies using the Newcastle Ottawa Scale (NOS).

**Additional file 4.** Proportions of 30-day readmissions after CABG in non-index hospitals.

**Additional file 5.** Systematic review on association between comorbidity indices and 30-day readmission rate after CABG.

**Additional file 6.** Forest plots showing individual meta-analysis for 16 patient-level factors (S1-S16 Figures).

## Data Availability

All data generated or analyzed in this study are included in this published article. Also, the references for all included studies are given.

## Abbreviations

CABG: Coronary artery bypass graft
CI: Confidence interval
HRRP: Hospital Readmissions Reduction Program
MI: Myocardial infraction
NOS: Newcastle-Ottawa Scale
OR: Odds ratio
PCI: Percutaneous coronary artery interventions
