# Supplementary material for: Patient and hospital factors associated with 30-day readmissions after coronary artery bypass graft (CABG) surgery: a systematic review and meta-analysis
Source: J Cardiothorac Surg. 2021 Jun 10;16:172. doi: 10.1186/s13019-021-01556-1 (PMC8194115; doi:10.1186/s13019-021-01556-1)
Supplement: Supplementary file 2 — Additional file 2. Search terms used for the systematic review. [file 13019_2021_1556_MOESM2_ESM.docx]

**S1 Table: Search terms used for the systematic review**

| **PubMed**  **Search interface: National Library of Medicine (NLM)** | |
| --- | --- |
| 1 | coronary artery bypass grafting[tiab] |
| 2 | cabg[tiab] |
| 3 | (coronary artery bypass grafting[tiab] OR cabg[tiab]) |
| 4 | hospital readmission[tiab] |
| 5 | readmission[tiab] |
| 6 | hospital admission[tiab] |
| 7 | ((hospital readmission[tiab] OR readmission[tiab]) OR hospital admission[tiab]) |
| 8 | (coronary artery bypass grafting[tiab] OR cabg[tiab]) AND ((hospital readmission[tiab] OR readmission[tiab]) OR hospital admission[tiab]) |
|  |  |
| **Embase**  **Search interface: Ovid** | |
| 1 | coronary artery bypass grafting.ti,ab. |
| 2 | cabg.ti,ab. |
| 3 | 1 or 2 |
| 4 | readmission*.ti,ab. |
| 5 | hospital readmission*.ti,ab. |
| 6 | hospital admission*.ti,ab. |
| 7 | 4 or 5 or 6 |
| 8 | 3 and 7 |
