# Supplementary material for: Patient and hospital factors associated with 30-day readmissions after coronary artery bypass graft (CABG) surgery: a systematic review and meta-analysis
Source: J Cardiothorac Surg. 2021 Jun 10;16:172. doi: 10.1186/s13019-021-01556-1 (PMC8194115; doi:10.1186/s13019-021-01556-1)
Supplement: Supplementary file 3 — Additional file 3. Quality assessment of included studies using the Newcastle Ottawa Scale (NOS). [file 13019_2021_1556_MOESM3_ESM.docx]

**S2 Table: Quality assessment of included studies using the Newcastle Ottawa Scale (NOS)**

| **Author's last name (Year), Country** | **Data source (Study period)** | **NOS** |
| --- | --- | --- |
| Alkhouli (2019), USA | National Readmissions Database (2015-2016) | 9 |
| Anderson (2016), USA | California Coronary Artery Bypass Grafting Outcomes Reporting Program (2011-2012) | 8 |
| Angraal (2018), USA | Medicare fee-for-service inpatient claims data (1999-2004) | 7 |
| Auerbach (2009), USA | Clinical data from multiple centres in North Carolina (2003-2005) | 7 |
| Barnett (2018), USA | Clinical data from multiple centres (2008-2011) | 7 |
| Benuzillo (2018), USA | Clinical data from multiple centres (2010-2014) | 8 |
| Bianco (2019), USA | Pennsylvania Health Care Cost Containment Council databases (2014-2016) | 7 |
| Bianco (2019), USA | Clinical data from a single centre (2011-2017) | 6 |
| Blackledge (2009), UK | Hospital inpatient data (1995-2004) | 6 |
| Brooke (2015), USA | Medicare and Medicaid beneficiary data (2001-2011) | 9 |
| Case (2019), USA | Clinical data from a single centre (2011-2017) | 6 |
| Chan (2020), USA | Clinical data from a single centre (2011-2017) | 6 |
| Chen (2015), USA | Patient discharge database (2011) | 6 |
| Cho (2019), USA | Clinical data from a single centre (2013-2016) | 6 |
| Connolly (2018), USA | Florida, California, New York, Maryland, and Kentucky State Inpatient Databases (2007–2014) | 7 |
| Deo (2019), USA | National readmission database (2014) | 9 |
| Fanari (2017), USA | Clinical data from multiple centres (2010-2013) | 7 |
| Feng (2018), USA | California, Florida, and New York State Inpatient Databases (2007-2011) | 7 |
| Fox (2013), USA | California State Inpatient and Emergency Department Databases (2005-2009) | 7 |
| Girotti (2014), USA | National Medicare beneficiaries database (2006-2008) | 8 |
| Gurram (2019), India | Clinical data from a single centre (2015-2016) | 6 |
| Hannan (2003), USA | New York state inpatient database (1999) | 7 |
| Hannan (2011), USA | New York state inpatient database (2005-2007) | 7 |
| Hirji (2020), USA | National readmission database (2010-2015) | 9 |
| Hwang (2007), USA | 5% sample of Medicare beneficiaries database (2001-2003) | 6 |
| Iribarne (2014), USA | Clinical data from multiple centres in the United States and Canada (2010) | 9 |
| Khuory (2019), USA | Nationwide readmission database (2010-2014) | 9 |
| Kim (2015), USA | Administrative claims database (2010-2012) | 7 |
| Koochmeshki (2013), Iran | Clinical data from a single centre (2004-2011) | 6 |
| Lancey (2014), USA | Society of Thoracic Surgeons (STS) compliant registry (2007-2011) | 7 |
| Li (2012), USA | California CABG outcomes reporting program (2009) | 8 |
| Li (2014), USA | California CABG outcomes reporting program (2010-2011) | 8 |
| Li (2015), USA | California CABG outcomes reporting program (2012) | 8 |
| McNeely (2017), USA | Medicare and Medicaid Services data (2000-2012) | 9 |
| Narain (2019), UK | Clinical data from a single centre (2012-2017) | 6 |
| O'Brien (2018), Australia | Australian and New Zealand Society of Cardiac and Thoracic Surgeons registry (2000-2012) | 8 |
| Price (2013), USA | New York Cardiac Surgery Reporting System (CSRS) (2006-2011) | 7 |
| Reis (2008), Brazil | Clinical data from a single centre (2006-2007) | 6 |
| Rosenblum (2019), USA | Clinical data from a single centre (2002-2017) | 6 |
| Saab (2013), Lebanon | Clinical data from a single centre (2010) | 6 |
| Saito (2019), Japan | Japan Cardiovascular Surgery Database (2015-2016) | 7 |
| Sargin (2016), Turkey | Clinical data from a single centre (2013) | 6 |
| Sedrakyan (2016), USA | Register data from New York and California states (2005-2011) | 8 |
| Shah (2019), USA | National readmission database (2013-2014) | 9 |
| Shahian (2014), USA | Society of Thoracic Surgeons National Database (2008-2010) | 8 |
| Shehata (2013), Canada | Clinical data from a single centre (2007-2009) | 6 |
| Slamowicz (2008), Australia | Victorian Admitted Episodes Dataset (1998-2003) | 7 |
| Stewart (2000), USA | Clinical data from a single centre (1997) | 6 |
| Tam (2018), Canada | CorHealth Ontario Cardiac Registry (2008-2016) | 7 |
| Trooboff (2019), USA | Clinical data from multiple centres (2008-2010) | 8 |
| Tsai (2013), USA | National Medicare data (2009-2010) | 8 |
| Tseng (2018), Taiwan | Health insurance database (2005) | 7 |
| Zywot (2018), USA | Hospital Readmission reduction database from California and New York (2006-2011) | 8 |
