# Supplementary material for: Patient and hospital factors associated with 30-day readmissions after coronary artery bypass graft (CABG) surgery: a systematic review and meta-analysis
Source: J Cardiothorac Surg. 2021 Jun 10;16:172. doi: 10.1186/s13019-021-01556-1 (PMC8194115; doi:10.1186/s13019-021-01556-1)
Supplement: Supplementary file 4 — Additional file 4. Proportions of 30-day readmissions after CABG in non-index hospitals. [file 13019_2021_1556_MOESM4_ESM.docx]

**Table S3: Proportions of 30-day readmissions after CABG in non-index hospitals**

| **Author (Year)** | **Country** | **Data source (Study period)** | **No. of CABG patients** | **No. of readmitted** | **% readmitted to non-index hospitals** |
| --- | --- | --- | --- | --- | --- |
| Brooke (2015) | USA | Medicare beneficiaries data (2001-2011) | 1502815 | 283131 within 30 days | 34.20% |
| Hirji (2020) | USA | National Readmission Database (2010-2015) | 844206 | 104803 within 30 days | 27.30% |
| Li (2012) | USA | California CABG clinical registry data (2009-2010) | 11823 | 1565 within 30 days | 34.60% |
