# Supplementary material for: Patient and hospital factors associated with 30-day readmissions after coronary artery bypass graft (CABG) surgery: a systematic review and meta-analysis
Source: J Cardiothorac Surg. 2021 Jun 10;16:172. doi: 10.1186/s13019-021-01556-1 (PMC8194115; doi:10.1186/s13019-021-01556-1)
Supplement: Supplementary file 5 — Additional file 5. Systematic review on association between comorbidity indices and 30-day readmission rate after CABG. [file 13019_2021_1556_MOESM5_ESM.docx]

**S4 Table: Systematic review on association between comorbidity indices and 30-day readmission rate after CABG**

| **Author (year)** | **Country (study period)** | **Comorbidity index** | **Findings** |
| --- | --- | --- | --- |
| Chen (2015) | USA (2011) | Charlson comorbidity index | 30-day readmission rate: 0 = 5.8% 1 = 7.6% 2 = 11.4% 3+ = 16.3% |
| Cho (2019) | USA (2013-2016) | Charlson comorbidity index | Per unit increase in the index was associated with increased rate of readmission (OR 1.11, 95% CI: 1.01-1.21) |
| Khuory (2019) | USA (2010-2014) | Elixhauser Comorbidity Index | Mean index was 4.4 among those who were readmitted within 30 days after CABG vs 3.6 among those who were not readmitted. |
| Kim (2015) | USA | Charlson comorbidity index | Adjusted OR for Charlson Index ≥2 vs 0 was 1.37 (95% CI 1.33–1.42) |
| Shah (2019) | USA (2013-2014) | Elixhauser Comorbidity Index | Compared to those with <4 Elixhauser comorbidities, those with >4 Elixhauser comorbidities were more likely to be readmitted (OR 1.20, 95% CI: 1.15-1.25). |
| Shehata (2013) | Canada (2007-2009) | Charlson comorbidity index | ORs for 30-day readmission:  0 = reference  1-2 = not significant  3-4 = 2.3 (1.4-3.7) 5 + = 2.1 (1.3-3.6) |
| Slamowicz (2008) | Australia (1998-2003) | Charlson comorbidity index | Adjusted OR for per unit increase in Charlson comorbidity index was 1.18 (1.11-1.24) |
| Tam (2018) | Canada (2008-2016) | Charlson comorbidity index | Mean index among those who were readmitted and were not readmitted was 1.6 and 1.3, respectively. |
| Tseng (2018) | Taiwan (2005) | Charlson comorbidity index | Mean index among those who were readmitted and were not readmitted was 1.2 and 0.9, respectively. |
