# Supplementary material for: Patient and hospital factors associated with 30-day readmissions after coronary artery bypass graft (CABG) surgery: a systematic review and meta-analysis
Source: J Cardiothorac Surg. 2021 Jun 10;16:172. doi: 10.1186/s13019-021-01556-1 (PMC8194115; doi:10.1186/s13019-021-01556-1)
Supplement: Supplementary file 6 — Additional file 6. Forest plots showing individual meta-analysis for 16 patient-level factors (S1-S16 Figures). [file 13019_2021_1556_MOESM6_ESM.docx]

**Individual meta-analysis for each variable presented in Fig 5.**


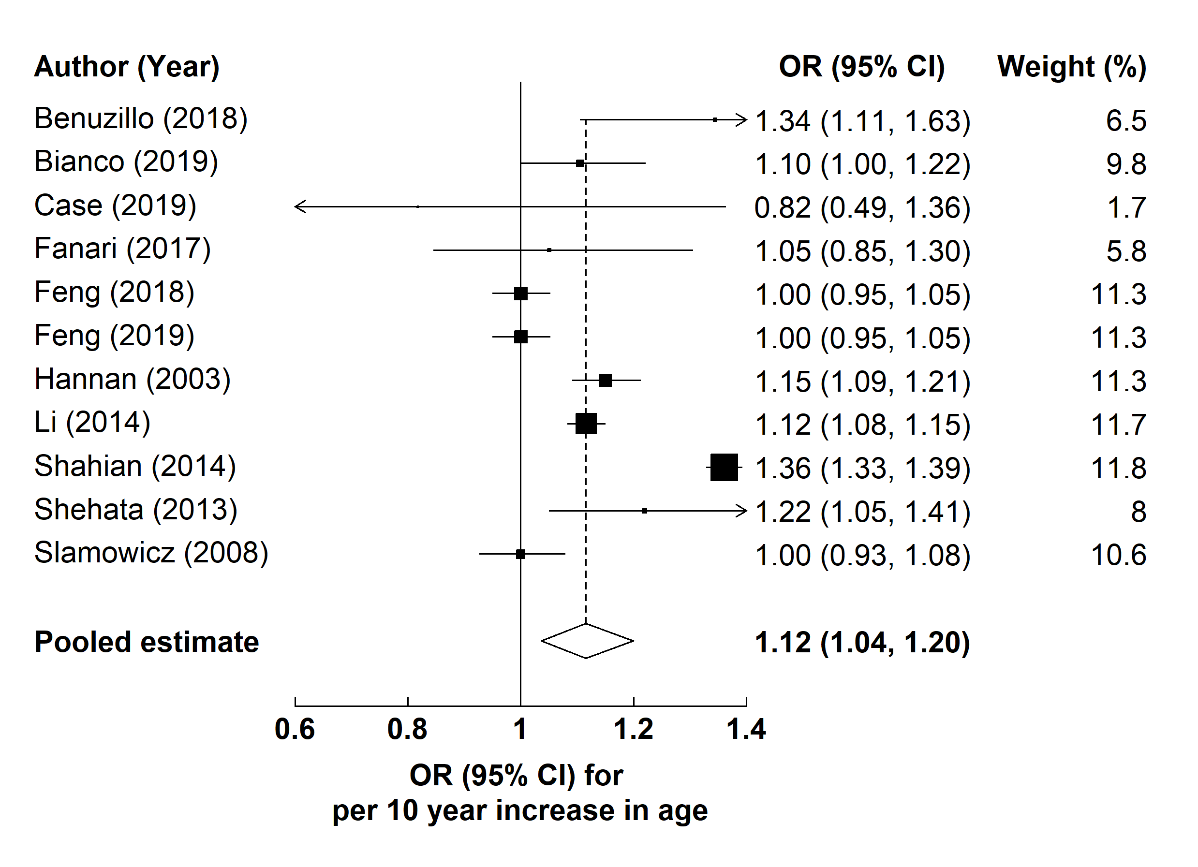


**Fig S1: Random effect meta-analysis for association between age (per 10-year increase) and 30-day all-cause readmission after CABG.** Individual study-specific odds ratios (ORs) and their 95% CIs are indicated by the black squares and the horizontal lines, respectively. The size of the black squares corresponds to the inverse of variance of the study-specific estimates included in the meta-analysis. The centre of the diamond indicates the pooled OR and the width of it indicates corresponding 95% CI.


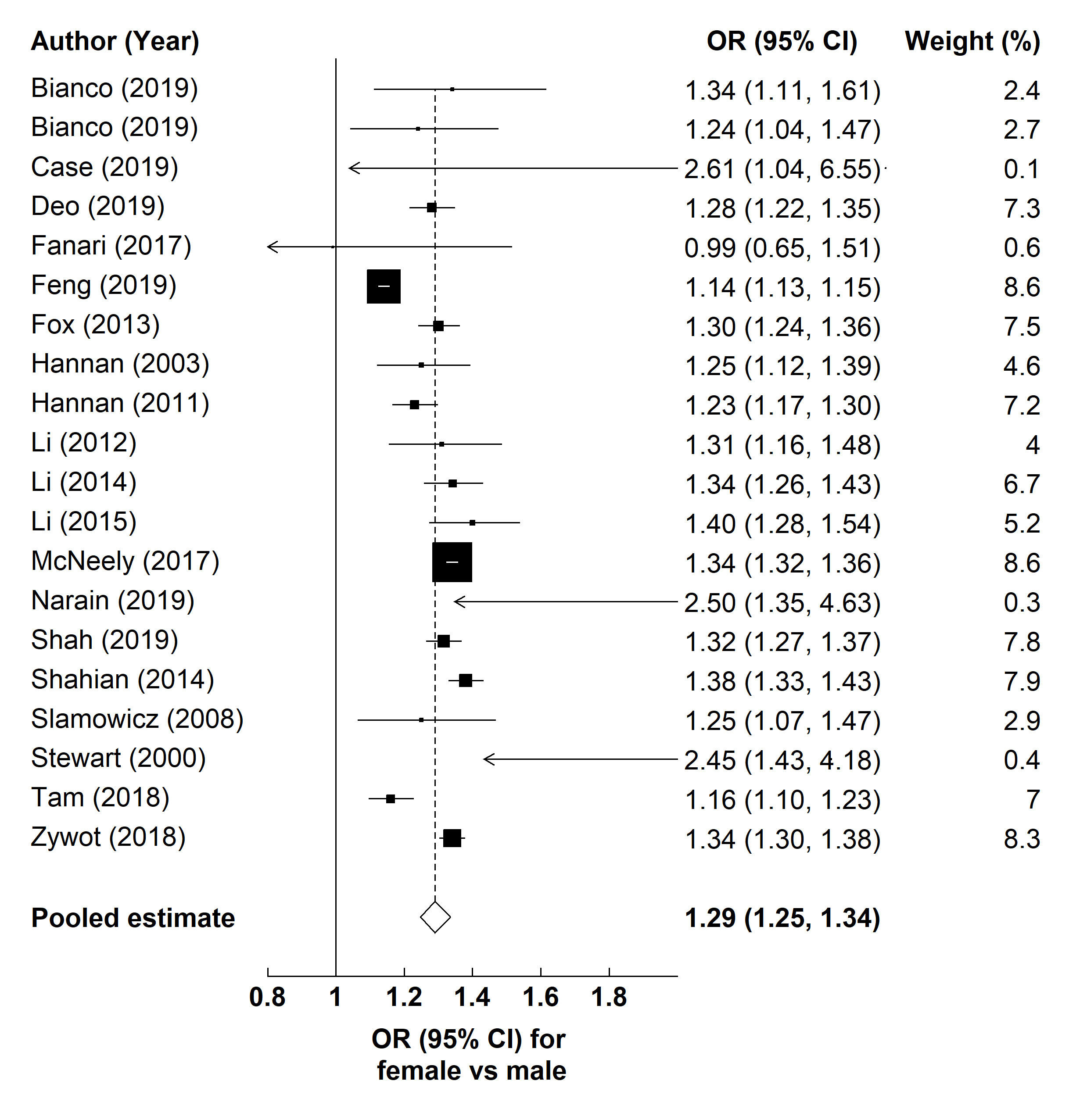


**Fig S2: Random effect meta-analysis for association between female sex and 30-day all-cause readmission after CABG.** Individual study-specific odds ratios (ORs) and their 95% CIs are indicated by the black squares and the horizontal lines, respectively. The size of the black squares corresponds to the inverse of variance of the study-specific estimates included in the meta-analysis. The centre of the diamond indicates the pooled OR and the width of it indicates corresponding 95% CI.


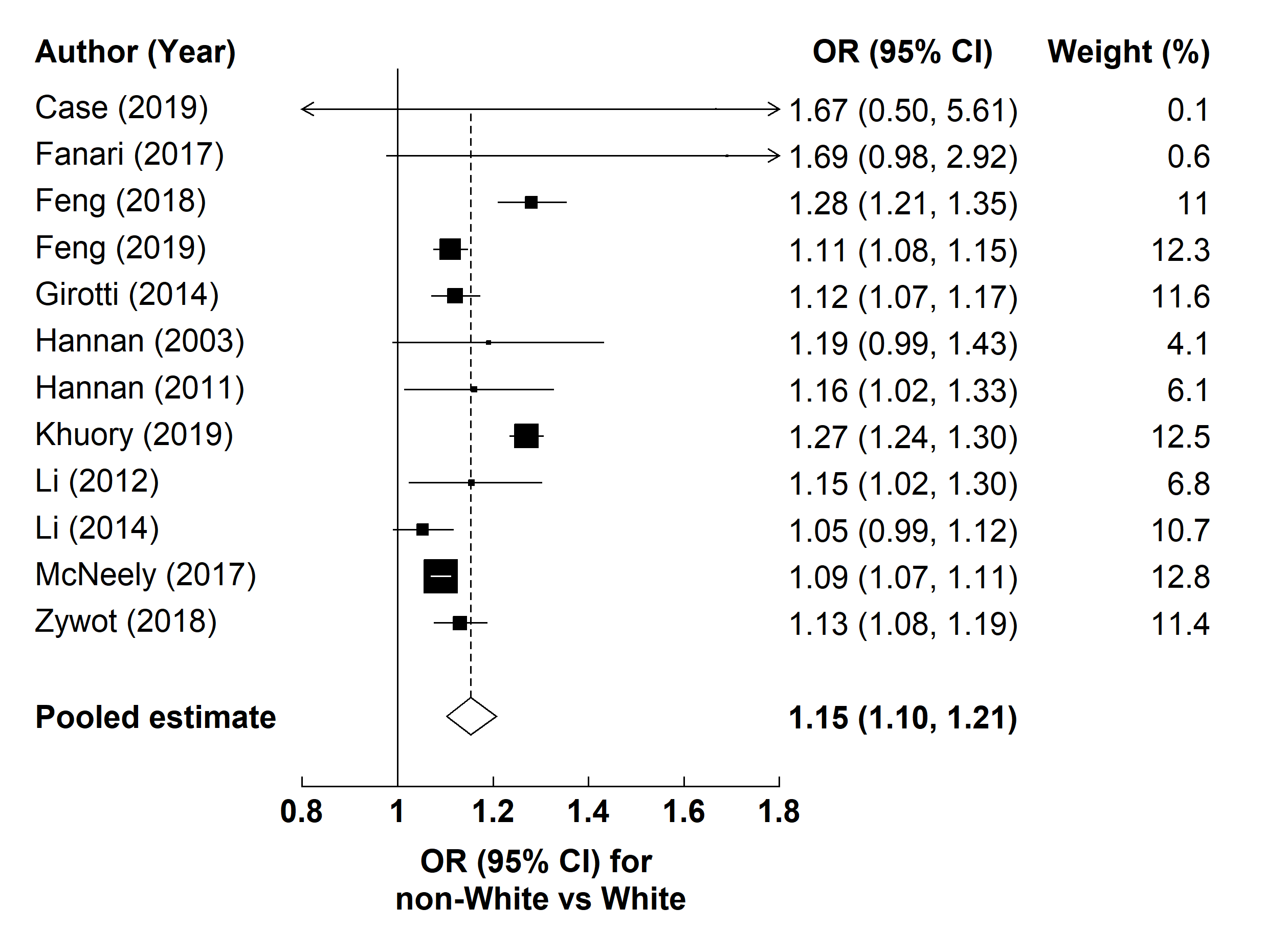


**Fig S3: Random effect meta-analysis for association between race (White vs non-White) and 30-day all-cause readmission after CABG.** Individual study-specific odds ratios (ORs) and their 95% CIs are indicated by the black squares and the horizontal lines, respectively. The size of the black squares corresponds to the inverse of variance of the study-specific estimates included in the meta-analysis. The centre of the diamond indicates the pooled OR and the width of it indicates corresponding 95% CI.


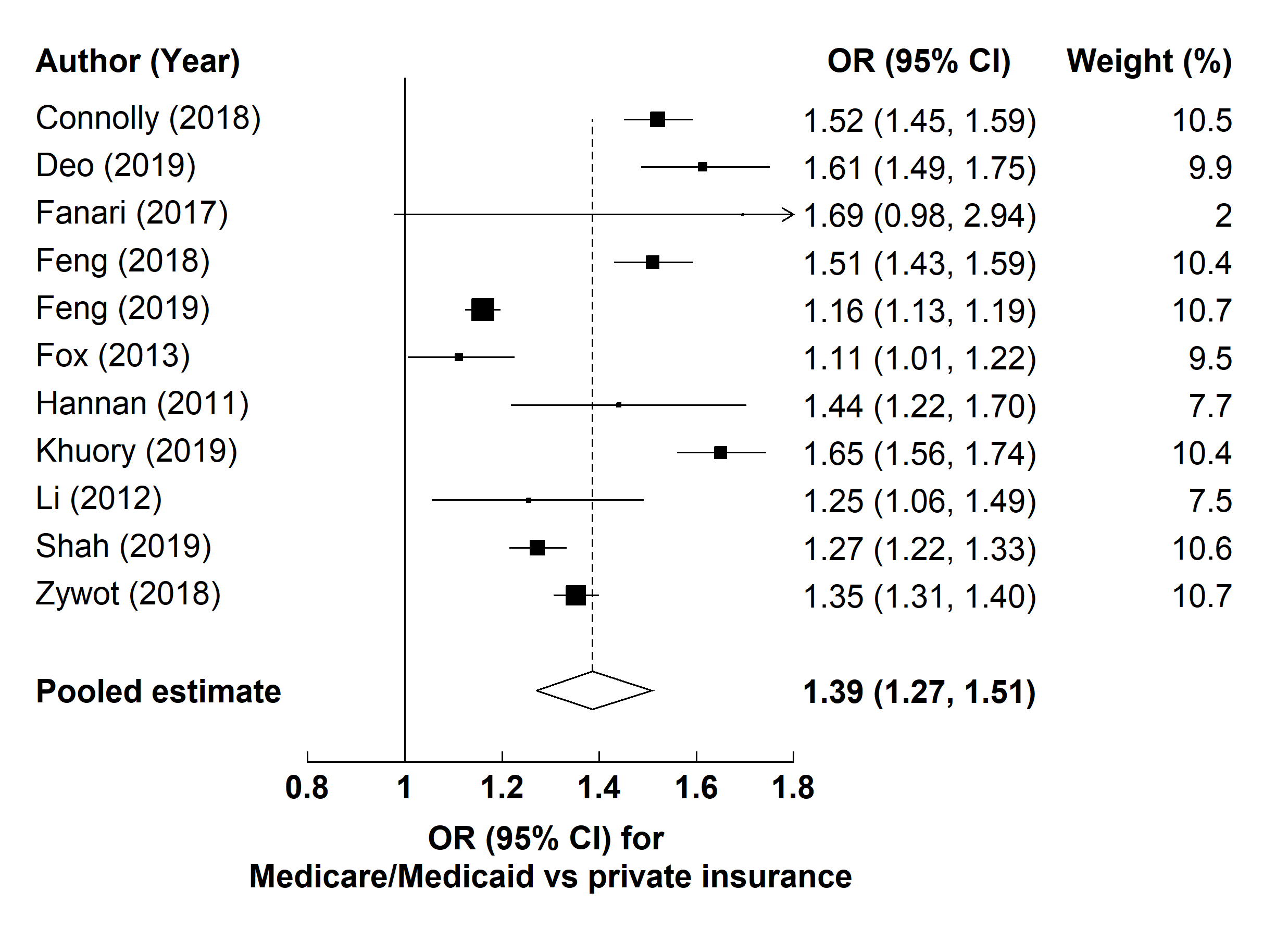


**Fig S4: Random effect meta-analysis for association between insurance (Medicare/Medicaid vs private) and 30-day all-cause readmission after CABG.** Individual study-specific odds ratios (ORs) and their 95% CIs are indicated by the black squares and the horizontal lines, respectively. The size of the black squares corresponds to the inverse of variance of the study-specific estimates included in the meta-analysis. The centre of the diamond indicates the pooled OR and the width of it indicates corresponding 95% CI.


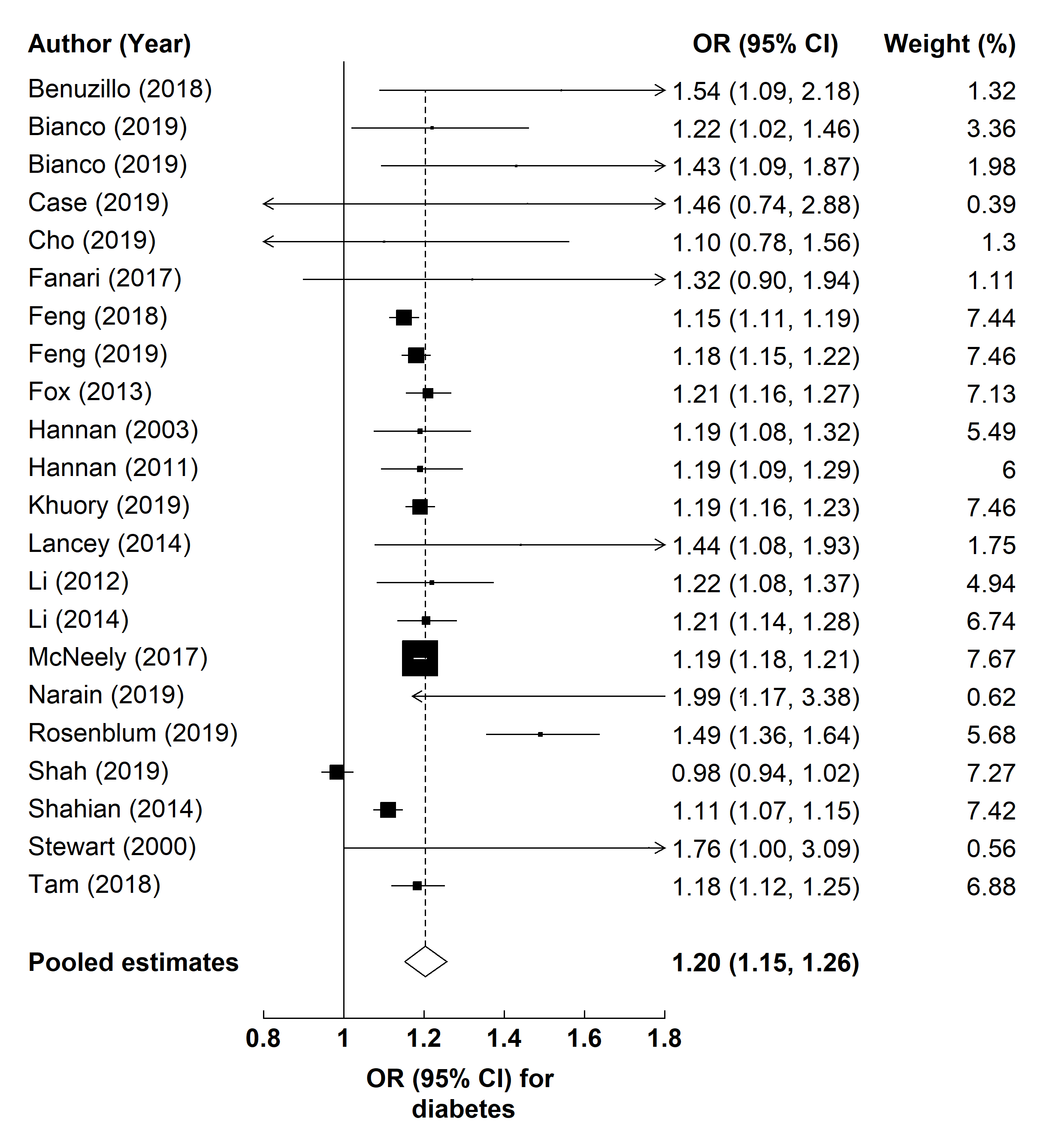


**Fig S5: Random effect meta-analysis for association between diabetes and 30-day all-cause readmission after CABG.** Individual study-specific odds ratios (ORs) and their 95% CIs are indicated by the black squares and the horizontal lines, respectively. The size of the black squares corresponds to the inverse of variance of the study-specific estimates included in the meta-analysis. The centre of the diamond indicates the pooled OR and the width of it indicates corresponding 95% CI.


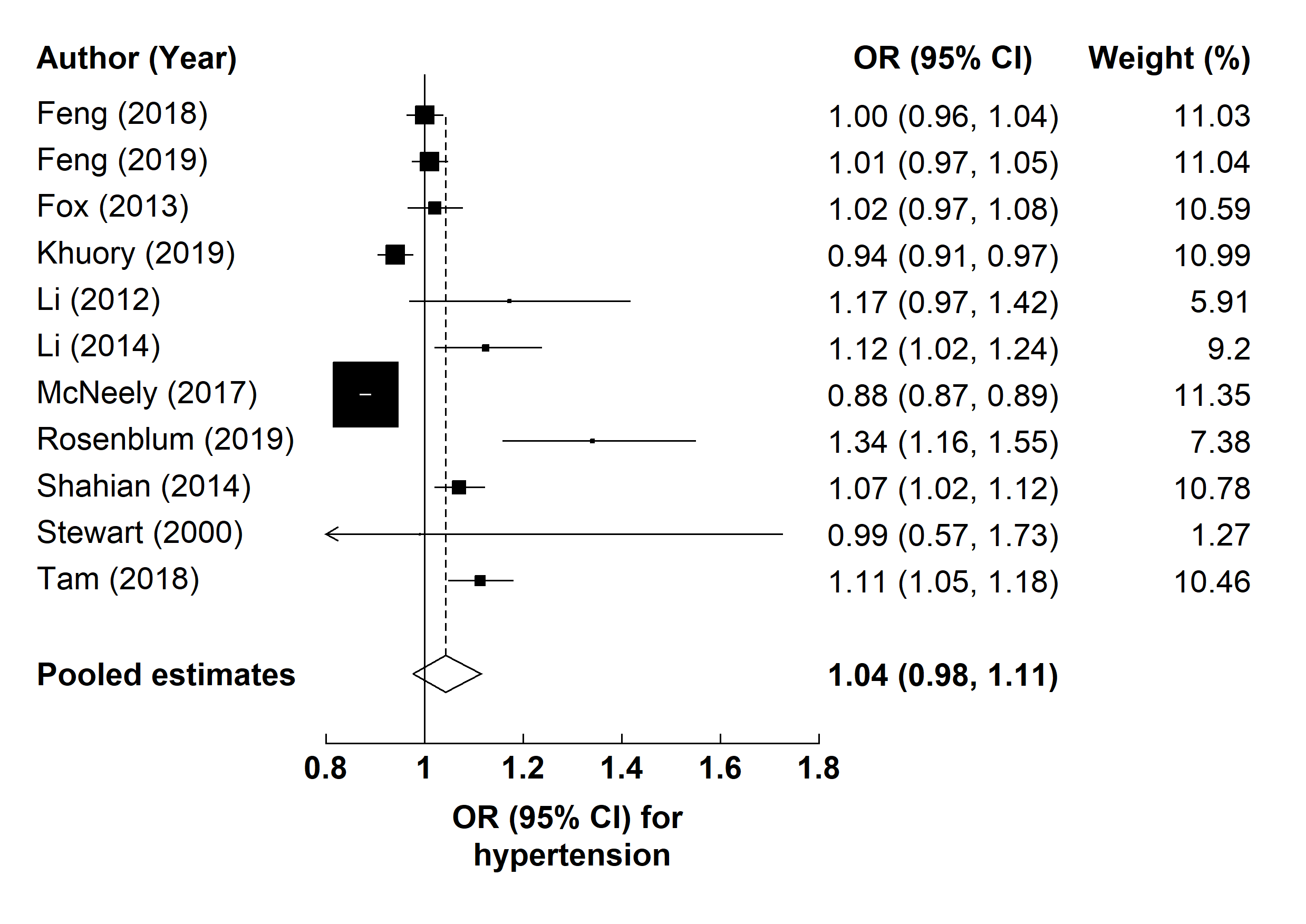


**Fig S6: Random effect meta-analysis for association between hypertension and 30-day all-cause readmission after CABG.** Individual study-specific odds ratios (ORs) and their 95% CIs are indicated by the black squares and the horizontal lines, respectively. The size of the black squares corresponds to the inverse of variance of the study-specific estimates included in the meta-analysis. The centre of the diamond indicates the pooled OR and the width of it indicates corresponding 95% CI.


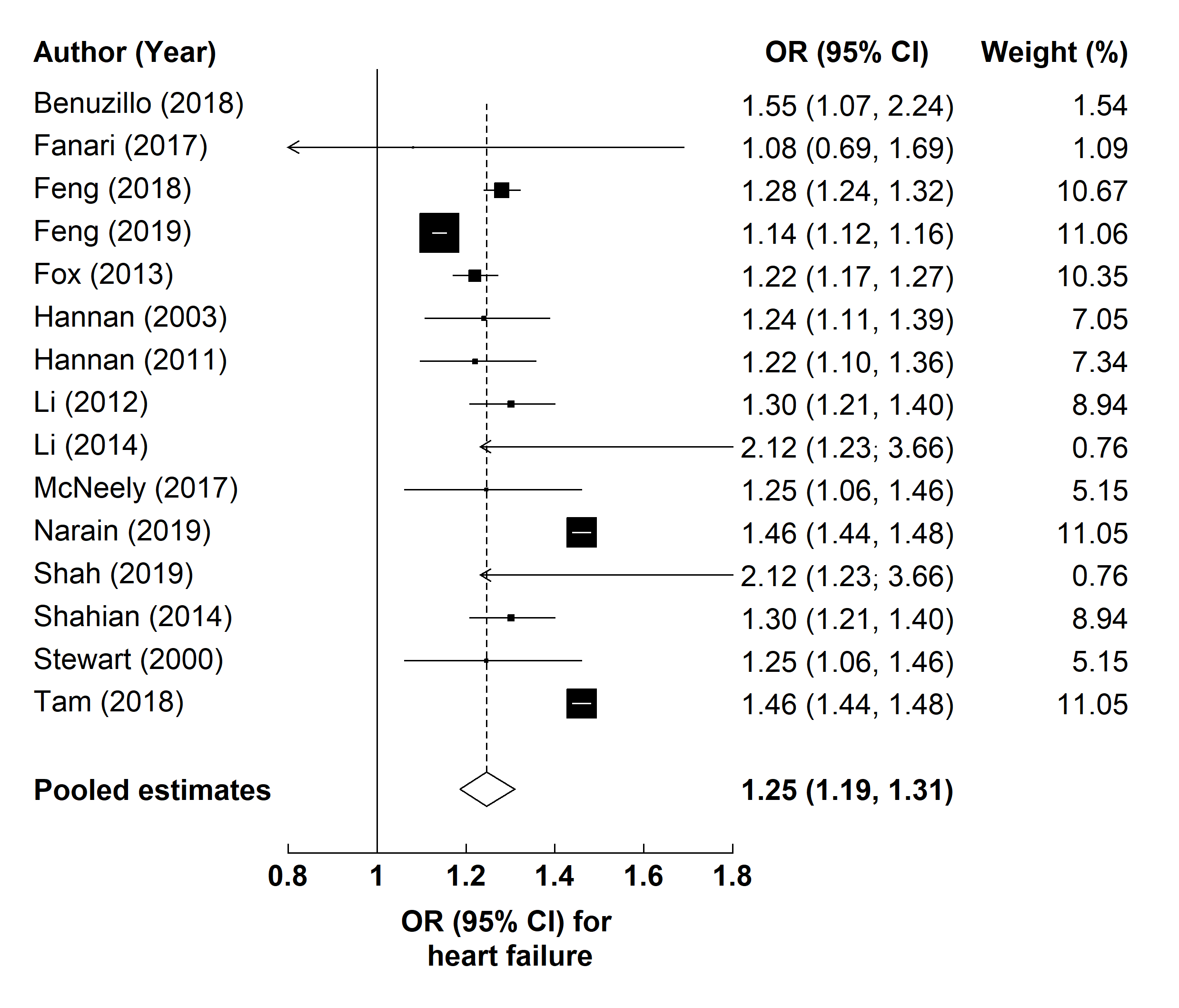


**Fig S7: Random effect meta-analysis for association between heart failure and 30-day all-cause readmission after CABG.** Individual study-specific odds ratios (ORs) and their 95% CIs are indicated by the black squares and the horizontal lines, respectively. The size of the black squares corresponds to the inverse of variance of the study-specific estimates included in the meta-analysis. The centre of the diamond indicates the pooled OR and the width of it indicates corresponding 95% CI.


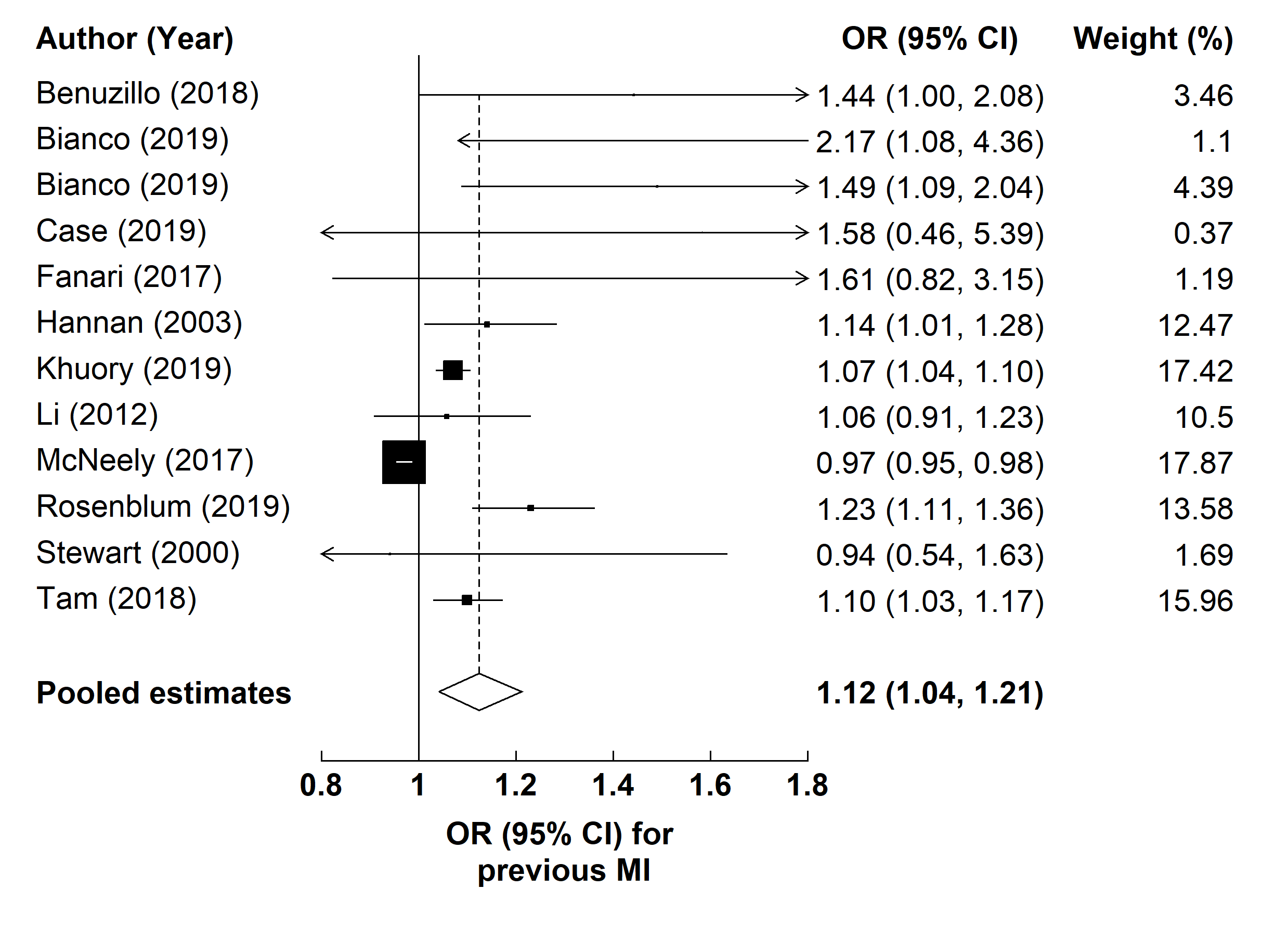


**Fig S8: Random effect meta-analysis for association between previous myocardial infraction and 30-day all-cause readmission after CABG.** Individual study-specific odds ratios (ORs) and their 95% CIs are indicated by the black squares and the horizontal lines, respectively. The size of the black squares corresponds to the inverse of variance of the study-specific estimates included in the meta-analysis. The centre of the diamond indicates the pooled OR and the width of it indicates corresponding 95% CI.


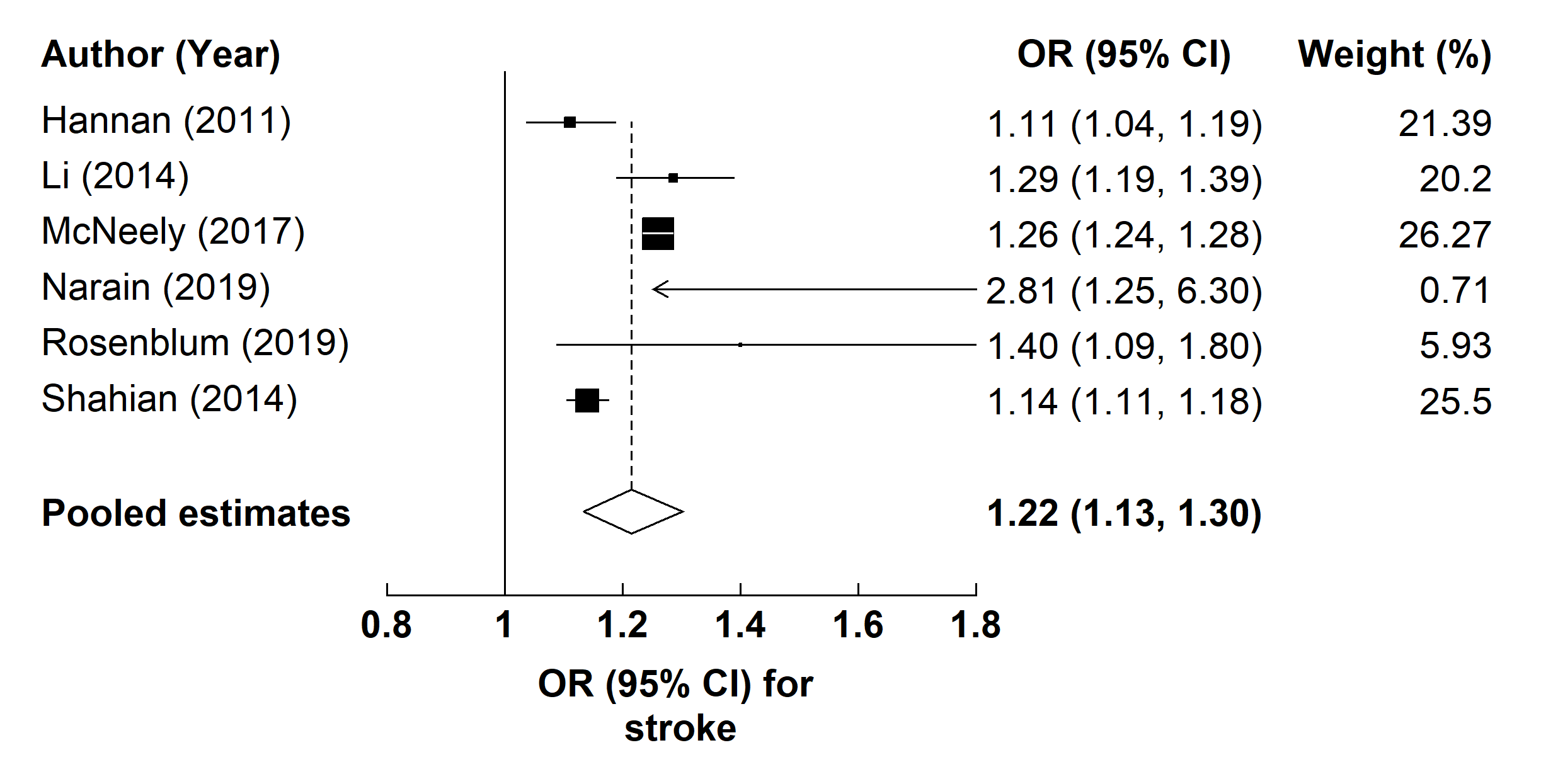


**Fig S9: Random effect meta-analysis for association between stroke and 30-day all-cause readmission after CABG.** Individual study-specific odds ratios (ORs) and their 95% CIs are indicated by the black squares and the horizontal lines, respectively. The size of the black squares corresponds to the inverse of variance of the study-specific estimates included in the meta-analysis. The centre of the diamond indicates the pooled OR and the width of it indicates corresponding 95% CI.


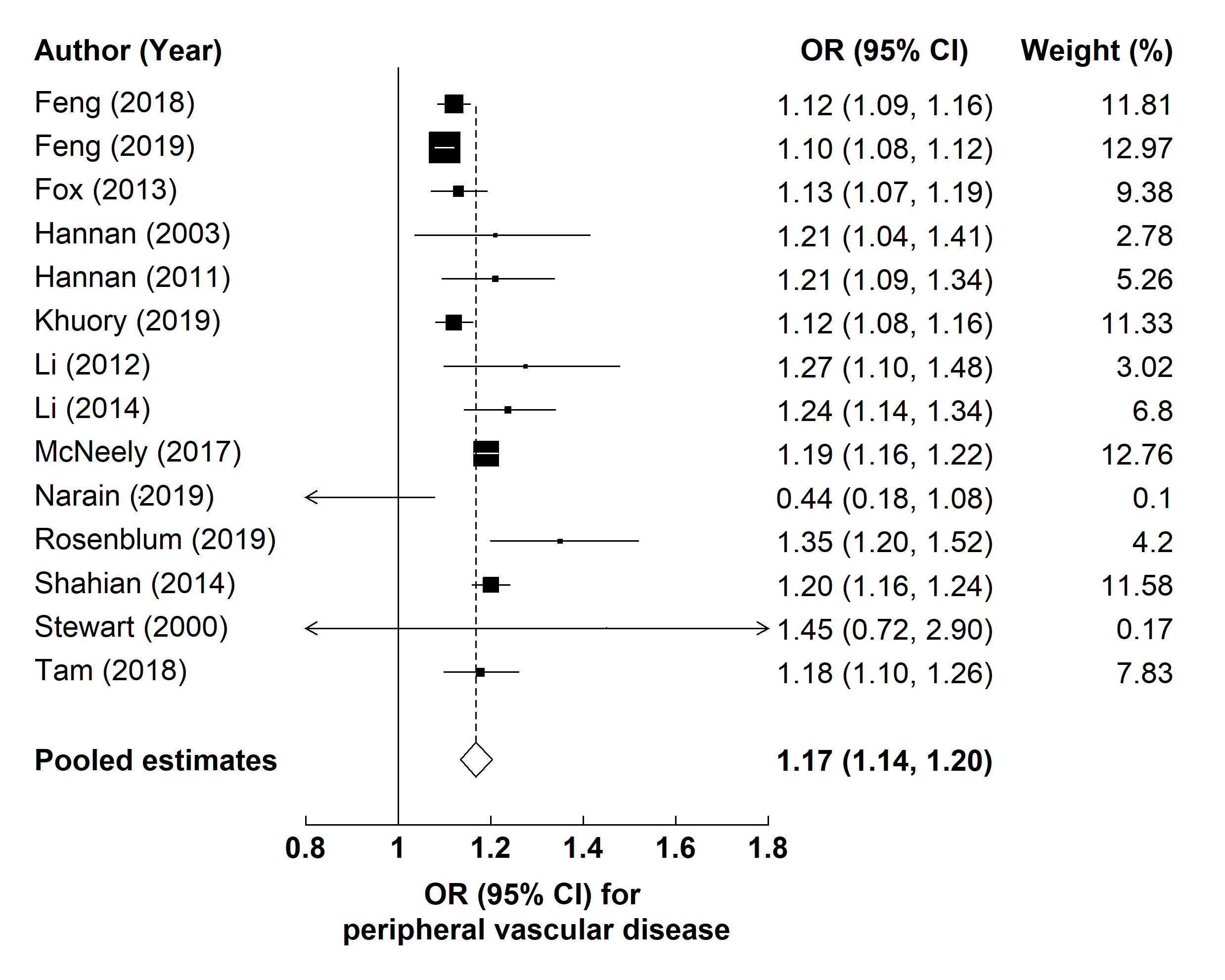


**Fig S10: Random effect meta-analysis for association between peripheral vascular disease and 30-day all-cause readmission after CABG.** Individual study-specific odds ratios (ORs) and their 95% CIs are indicated by the black squares and the horizontal lines, respectively. The size of the black squares corresponds to the inverse of variance of the study-specific estimates included in the meta-analysis. The centre of the diamond indicates the pooled OR and the width of it indicates corresponding 95% CI.


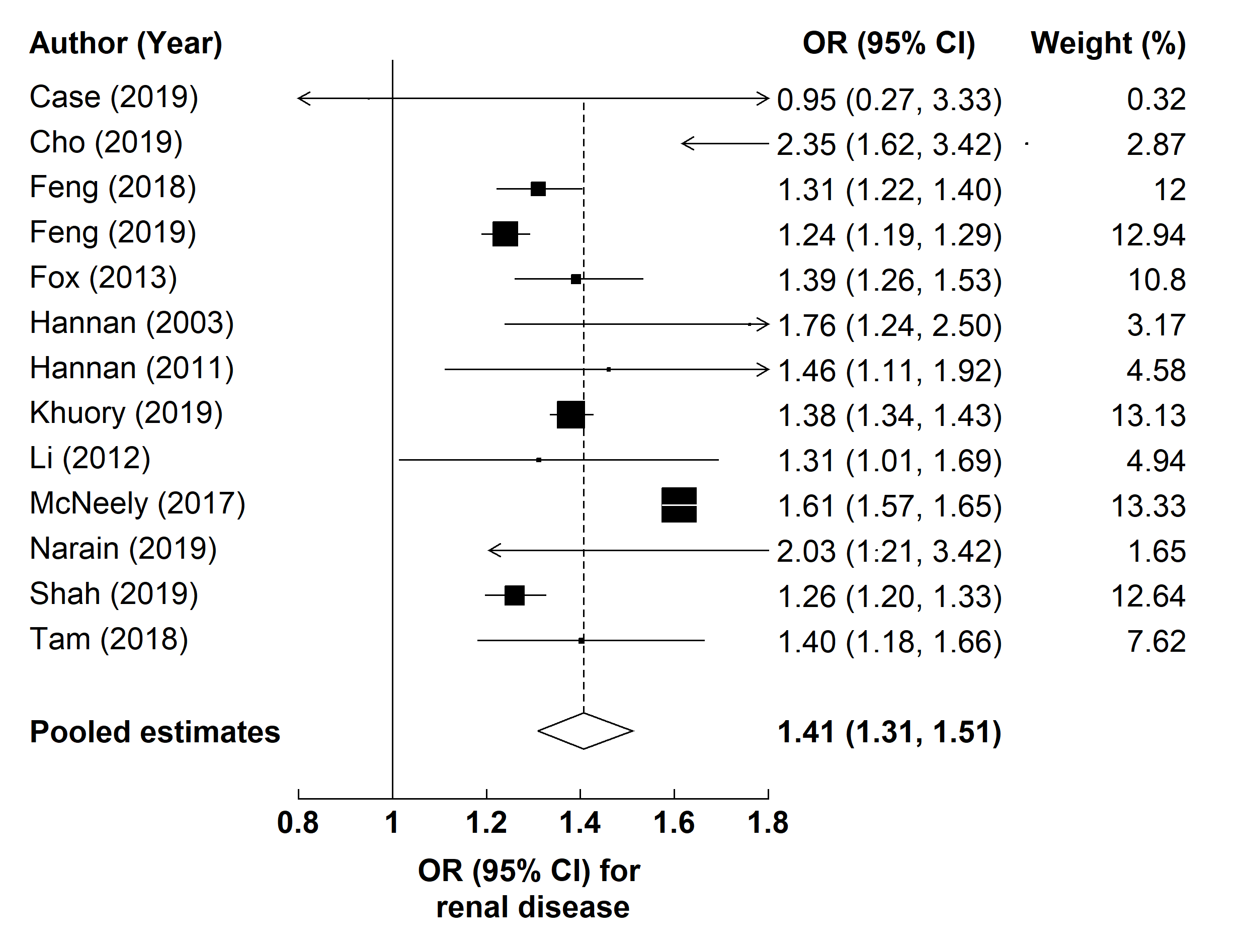


**Fig S11: Random effect meta-analysis for association between renal disease and 30-day all-cause readmission after CABG.** Individual study-specific odds ratios (ORs) and their 95% CIs are indicated by the black squares and the horizontal lines, respectively. The size of the black squares corresponds to the inverse of variance of the study-specific estimates included in the meta-analysis. The centre of the diamond indicates the pooled OR and the width of it indicates corresponding 95% CI.


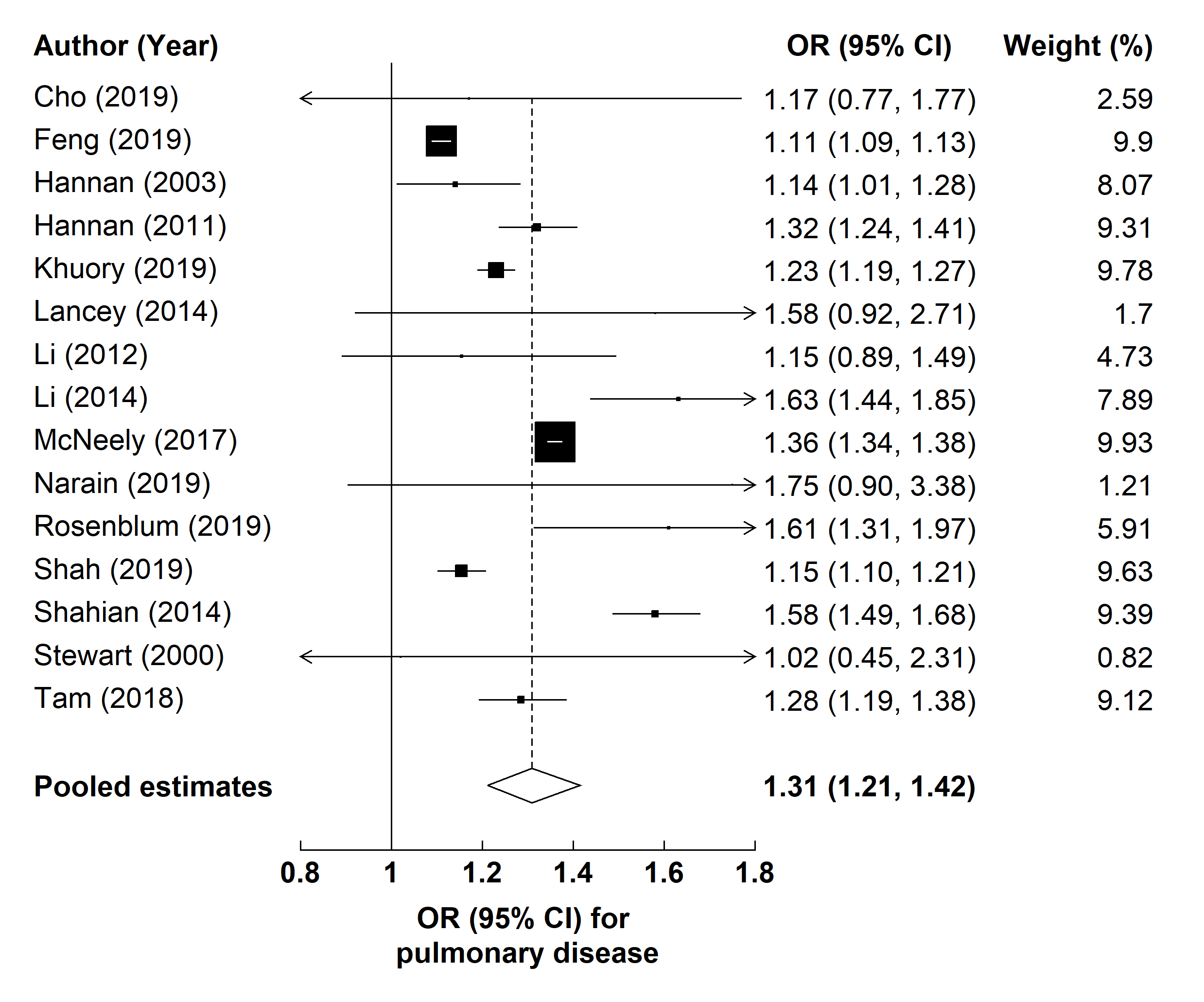


**Fig S12: Random effect meta-analysis for association between pulmonary disease and 30-day all-cause readmission after CABG.** Individual study-specific odds ratios (ORs) and their 95% CIs are indicated by the black squares and the horizontal lines, respectively. The size of the black squares corresponds to the inverse of variance of the study-specific estimates included in the meta-analysis. The centre of the diamond indicates the pooled OR and the width of it indicates corresponding 95% CI.


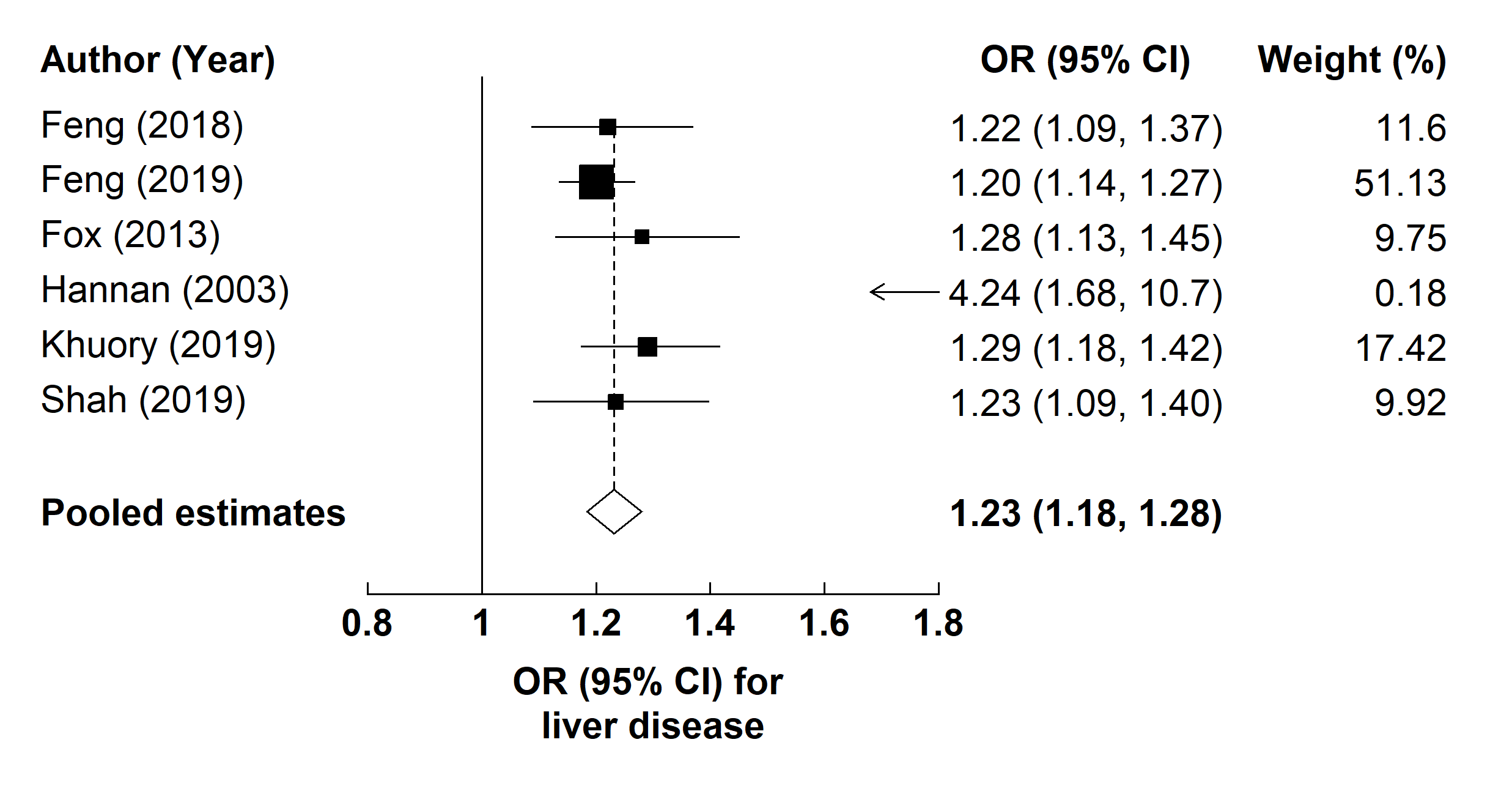


**Fig S13: Random effect meta-analysis for association between liver disease and 30-day all-cause readmission after CABG.** Individual study-specific odds ratios (ORs) and their 95% CIs are indicated by the black squares and the horizontal lines, respectively. The size of the black squares corresponds to the inverse of variance of the study-specific estimates included in the meta-analysis. The centre of the diamond indicates the pooled OR and the width of it indicates corresponding 95% CI.


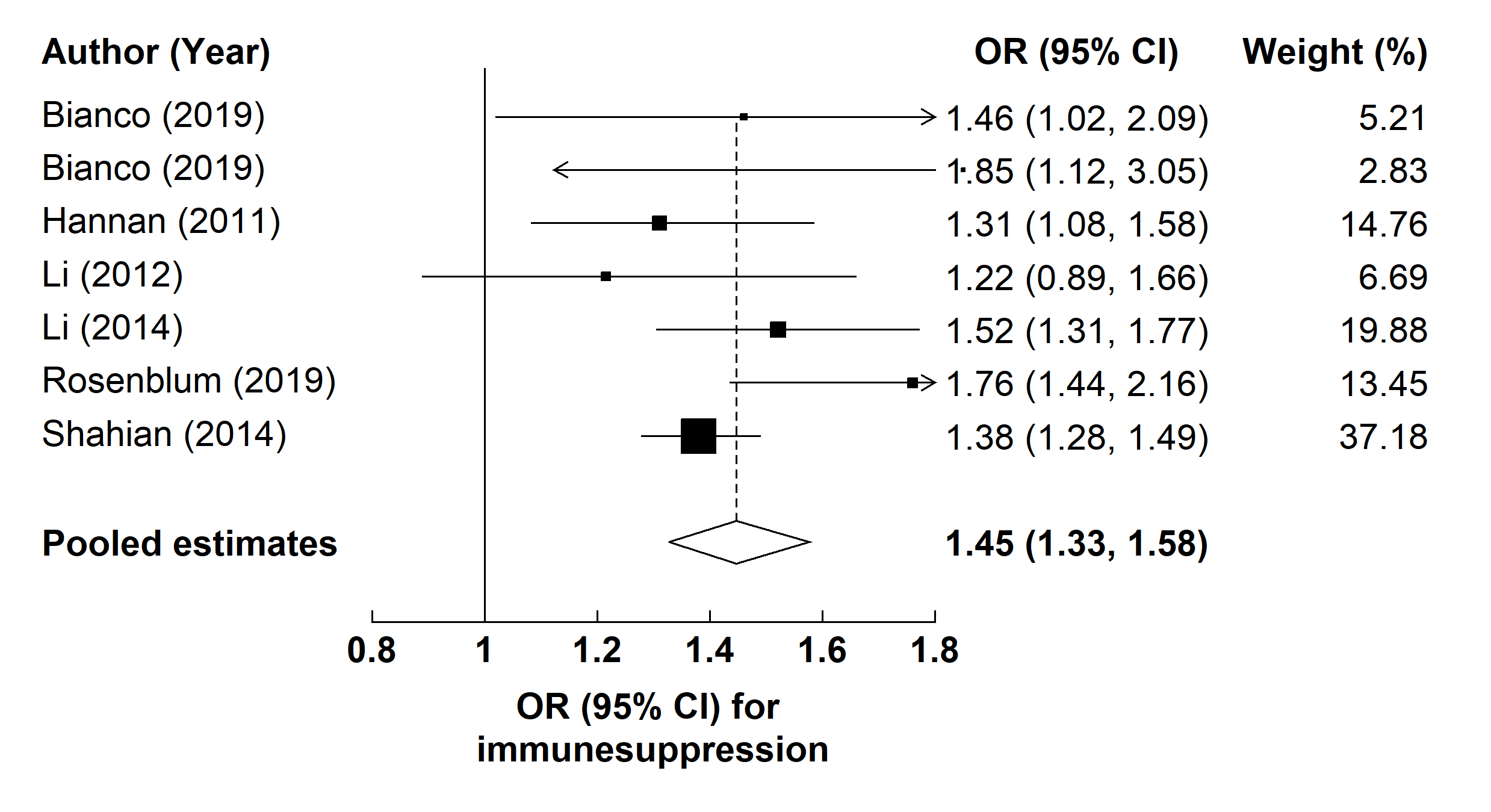


**Fig S14: Random effect meta-analysis for association between immunosuppression and 30-day all-cause readmission after CABG.** Individual study-specific odds ratios (ORs) and their 95% CIs are indicated by the black squares and the horizontal lines, respectively. The size of the black squares corresponds to the inverse of variance of the study-specific estimates included in the meta-analysis. The centre of the diamond indicates the pooled OR and the width of it indicates corresponding 95% CI.


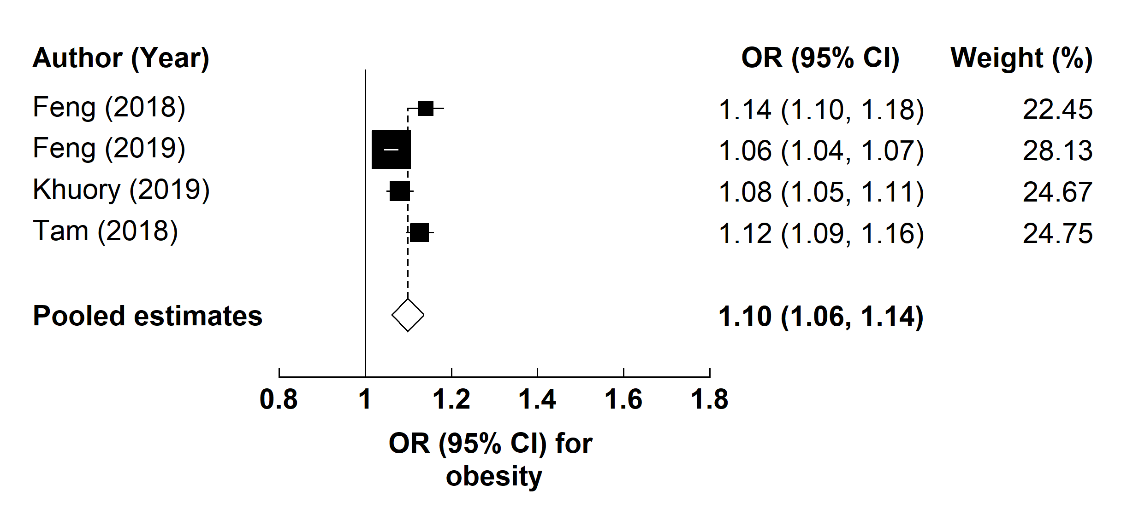


**Fig S15: Random effect meta-analysis for association between obesity and 30-day all-cause readmission after CABG.** Individual study-specific odds ratios (ORs) and their 95% CIs are indicated by the black squares and the horizontal lines, respectively. The size of the black squares corresponds to the inverse of variance of the study-specific estimates included in the meta-analysis. The centre of the diamond indicates the pooled OR and the width of it indicates corresponding 95% CI.


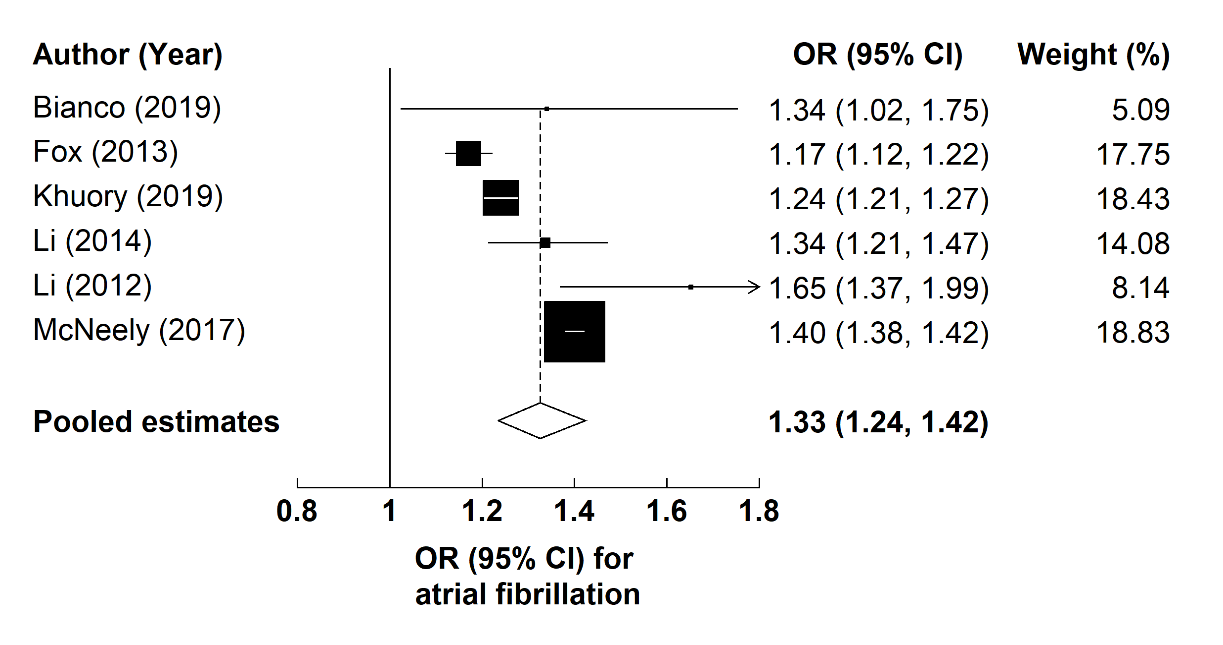


**Fig S16: Random effect meta-analysis for association between atrial fibrillation and 30-day all-cause readmission after CABG.** Individual study-specific odds ratios (ORs) and their 95% CIs are indicated by the black squares and the horizontal lines, respectively. The size of the black squares corresponds to the inverse of variance of the study-specific estimates included in the meta-analysis. The centre of the diamond indicates the pooled OR and the width of it indicates corresponding 95% CI.
